# EGFR ligand Angiogenin predicts response to ALK5 inhibition in pancreatic cancer via a TNF-α paracrine axis in tumor-associated macrophages

**DOI:** 10.1038/s41388-026-03774-0

**Published:** 2026-04-14

**Authors:** Silvia Pietrobono, Veronica De Vita, Domenico Mangiameli, Antonino Aparo, Eleonora San Lorenzo, Alice Bonato, Monica Bertolini, Enza Scarlato, Simona Casalino, Alberto Quinzii, Camilla Zecchetto, Davide Melisi

**Affiliations:** 1 Department of Medicine, Digestive Molecular Clinical Oncology Research Unit, University of Verona, Verona, Italy; 2 Research Center LURM (Interdepartmental Laboratory of Medical Research), University of Verona, Verona, Italy; 3 Investigational Cancer Therapeutics Clinical Unit, Azienda Ospedaliera Universitaria Integrata, Verona, Italy

**Keywords:** Predictive markers, Pancreatic cancer

## Abstract

Transforming growth factor-β (TGFβ) receptor ALK5 inhibition has shown promise in pancreatic ductal adenocarcinoma (PDAC), but predictive biomarkers remain undefined. We identify angiogenin (ANG) as a negative prognostic yet positive predictive biomarker for ALK5 inhibition combined with chemotherapy. In the randomized phase II H9H-MC-JBAJ trial, high baseline ANG predicted poor survival with gemcitabine alone but significant benefit from galunisertib addition. Mechanistic studies revealed that tumor-derived ANG binds epidermal growth factor receptor (EGFR) on tumor-associated macrophages (TAMs), activating RhoA-dependent cytoskeletal remodeling and autocrine ALK5/TGFβ signaling. This drives M2-like polarization and Smad2-mediated transcription of tumor necrosis factor-α (Tnf-α), which activates Nf-κB in neighboring tumor cells, conferring chemoresistance. ALK5 inhibition suppressed TAM-derived Tnf-α, reduced M2 polarization, prevented Nf-κB activation, and restored chemosensitivity in ANG-high models. Clinically, elevated ANG correlated with systemic TNF-α, and galunisertib reduced TNF-α exclusively in ANG-high patients, with reductions associated with markedly improved survival. These findings define an ANG–EGFR–TGFβ–TNF-α axis in TAMs as a stromal driver of PDAC chemoresistance, and provide a mechanistic rationale for the development of combination strategies targeting ALK5 signaling in ANG-high PDAC patients.

## Introduction

Pancreatic ductal adenocarcinoma (PDAC) continues to have the most severe prognosis among solid tumors, with the overall 5-year relative survival rate remaining low at just 13% [1, 2]. The dismal prognosis for PDAC patients is largely due to the limited efficacy of currently approved chemotherapy options. Furthermore, molecular targeted therapies, often tested in unselected PDAC populations without biomarker-based stratification, have also shown limited success [3].

Activin receptor-like kinase 5 (ALK5), also known as Transforming Growth Factor Beta (TGFβ) Receptor Type I (TGFβRI), is a key mediator of the TGFβ signaling pathway, which plays a complex, context-dependent role in PDAC [4]. While TGFβ signaling maintains cellular homeostasis in normal tissues, its tumor-suppressive effects are often bypassed in genetically unstable cancer cells, enabling TGFβ to promote tumor growth, epithelial-to-mesenchymal transition (EMT), and metastasis [5]. Although mutations in ALK5 are rare in PDAC, its signaling is frequently overactivated due to overexpression of TGFβ ligands and increased ALK5 expression in tumor cells or the surrounding stroma [6]. TGFβ signaling is a major driver of fibrosis and desmoplasia in PDAC through the activation of pancreatic stellate cells and cancer-associated fibroblasts, and it contributes to immune exclusion by enhancing TGFβ-mediated suppression of CD8 + T cells and dendritic cells [7].

Numerous ALK5 inhibitors have been developed, tested in preclinical murine models, and advanced to various clinical trials [4]. However, many of these agents have been discontinued during clinical development, likely due to being tested in unselected patient populations. We propose that the current approach to developing new experimental therapeutics for PDAC should be reconsidered, placing greater emphasis on the early identification of biological characteristics that could serve as biomarkers to guide patient selection.

Over the past decade, our research has contributed to advancing ALK5 inhibition as a therapeutic approach for PDAC, particularly in combination with chemotherapeutic [8–10] or immunotherapeutic agents [11]. In the phase 2b/randomized phase 2 H9H-MC-JBAJ trial involving patients with newly diagnosed unresectable PDAC, the addition of the ALK5 inhibitor galunisertib to gemcitabine demonstrated a positive trend in improving overall survival (OS) compared to gemcitabine alone [9]. Beyond clinical outcomes, the randomized nature of the study enabled extensive translational analyses on biological samples, leading to the identification of potential predictive biomarkers to guide patient selection for ALK5 inhibition [12].

Angiogenin (ANG, also known as RNase 5) is a secreted growth factor initially identified for its role in promoting the formation of new blood vessels. More recently, ANG secreted by tumor cells has been recognized as a key regulator in various pathological processes, influencing cell proliferation, survival, migration, invasion, and differentiation [13]. In the tumor microenvironment, secreted ANG can trigger a series of responses by binding to different receptors. Plexin-B2 was the first functional receptor for ANG identified in both normal and cancer cells [14]. More recently, ANG has been identified as a ligand for the Epidermal Growth Factor Receptor (EGFR) through a mechanism independent of its RNase catalytic activity [15]. ANG promotes tumorigenesis in vivo in an EGFR kinase activity-dependent manner, as evidenced by whole-transcriptome analysis, which reveals a similar pattern of transcriptional changes following treatment with ANG and EGF. Notably, ANG knockdown reduced sensitivity to the EGFR inhibitor erlotinib in PDAC models, while elevated plasma ANG levels were associated with increased sensitivity to this treatment in PDAC patients [15].

In this study, we identified ANG as a significant positive predictive biomarker for patients treated with the combination of the ALK5 inhibitor galunisertib and chemotherapy. We further investigated the molecular and cellular interactions between tumor cells and TAMs that underlie the remarkable response to ALK5 signaling pathway inhibition in PDAC, characterized by elevated ANG levels.

## Materials and methods

### Study population

This study focused on patients enrolled in phase 2 of the H9H-MC-JBAJ clinical trial, a global, two-part investigation assessing the oral administration of galunisertib in combination with gemcitabine. Inclusion and exclusion criteria are detailed in Supplemental Materials and Methods. All procedures involving human participants, tissues, or data were conducted in accordance with the principles of good clinical practice, applicable laws and regulations, the Council for International Organizations of Medical Sciences’ International Ethical Guidelines, and the Declaration of Helsinki. The local ethical review board approved the study. Written informed consent was obtained prior to participation.

### In vivo orthotopic transplantations

Five to six-week-old C57BL/6J female mice with body weights ranging from 21 to 25 g were purchased from Charles River Laboratories. Murine PDAC cells were resuspended in a PBS: Matrigel solution (1:1) at a concentration of 2.5 × 10^5^ cells per 40 μl/injection. On day 0, mice were anesthetized by exposure to isoflurane and injected orthotopically into the pancreas parenchyma. Treatments’ dosing and schedules are detailed in Supplemental Materials and Methods. Research involving animals was conducted in accordance with the relevant guidelines and regulations, and was approved by the Italian Ministry of Health (auth. 299/2022-PR, prot. C46F4.29).

### Statistical analysis

Data represent mean ± s.d. values calculated on at least three independent experiments. *P* values were calculated using Student’s *t* test (two groups) or one-way analysis of variance (ANOVA) with Dunnett’s or Tukey’s corrections (more than two groups). A two-tailed value of *p* < 0.05 was considered statistically significant. *,*p* < 0.05; **,*p* < 0.01, ***,*p* < 0.001. Overall survival (mOS) was estimated by using the Kaplan–Meier method, and a log-rank test was used to compare the results between groups. Statistical analyses were performed using GraphPad Prism version 9 and R version 4.2.2 (R Foundation for Statistical Computing. URL https://www.R-project.org/).

All other methods are available in the Supplementary Information section.

## Results

### Angiogenin is a negative prognostic but a positive predictive biomarker for response to ALK5 Inhibition in PDAC patients

**Fig. 1:**
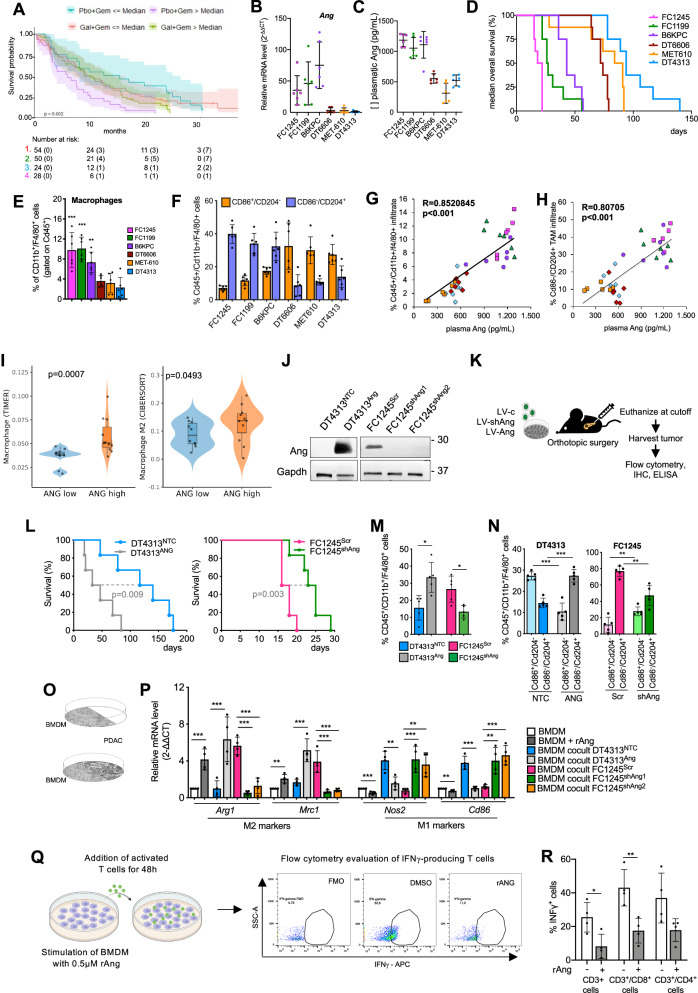
Angiogenin is a negative prognostic factor, as it sustains a protumor, M2-like phenotype in TAMs. **A** Kaplan–Meier estimates of overall survival (OS) by plasma levels of ANG, with 95% confidence bands and numbers at risk. Gal= galunisertib; Gem= gemcitabine; Pbo = placebo. **B** qPCR of *Ang* in orthotopic PDAC tumors from C57BL/6J mice. Data are expressed as mean ± s.d. (*n* = 6). **C** Plasma levels of Ang measured in C57BL/6 J mice bearing orthotopic PDAC tumors from 6 different murine PDAC cell lines using Enzyme-Linked Immunosorbent Assay (ELISA). Data are expressed as mean ± s.d. (*n* = 6). **D** Median overall survival (mOS) in C57BL/6J mice bearing orthotopic PDAC tumors (*n* = 8). Flow cytometry analysis of Cd45^+^/Cd11b^+^/F4/80^+^ tumor-associated macrophages (TAMs) (**E**) and their M1 (Cd86^+^/Cd204^-^)/M2 (Cd86^-^/Cd204^+^) polarization (**F**) in FC1245, FC1199, B6KPC, DT6606, MET-610 and DT4313 tumor samples. Data are expressed as mean ± s.d. (*n* = 6). Pearson’s correlation of secreted Ang with the infiltration of total TAMs (**G**) or with M2-polarized TAMs (**H**) in plasma from C57BL6/J PDAC models (*n* = 36). FC1245: pink; FC1199 green; B6KPC: violet; MET-610: orange; DT6606: red; DT4313: light blue. **I** Violin plots based on TCGA-PAAD data showing density distributions of TAMs (TIMER algorithm, left) or M2-TAMs (CIBERSORT algorithm, right) between ANG-high and ANG-low patients with metastatic PAAD. **J** Representative western blot of Ang in FC1245 and DT4313 transduced as indicated. Gapdh was used as a loading control. **K** Schematic representation of the in vivo orthotopic experiment. **L** mOS duration of C57BL6/J mice bearing orthotopic tumors from FC1245 or DT4313 transduced as indicated (*n* = 6). Flow cytometry analysis showing increased infiltration of Cd45^+^/Cd11b^+^/F4/80^+^ TAMs (**M**) and their M1(Cd45^+^/Cd11b^+^/F4/80^+^/Cd204^-^/Cd86^+^)/M2(Cd45^+^/Cd11b^+^/F4/80^+^/ Cd204^+^/Cd86^-^) skewing (**N**) in Ang-high FC1245^Scr^ and DT4313^Ang^ tumors compared with Ang-low FC1245^shAng^ or DT4313^NTC^ controls. Data are expressed as mean ± s.d. (*n* = 5). **O** Schematic representation of the co-culture technique used to culture BMDM macrophages with PDAC cell lines. **P** qPCR analysis of M1 (*Inos2*, *Cd86*) and M2 (*Arg1*, *Mrc1*) markers in BMDM left unstimulated or stimulated with rAng, as well as single cultures or co-cultured with FC1245 or DT4313 transduced as indicated. Data are shown as mean ± s.d (*n* = 4). **Q** Schematic representation of T cell suppression assay, with gating strategy. **R** Flow cytometry analysis of IFNγ expression in activated CD3^+^, CD8^+^ or CD4^+^ T cells when co-cultured for 48 h at a 1:5 ratio with BMDM, left unstimulated or pre-stimulated for 24 h with rAng (*n* = 4). *P* values were calculated using two-tailed unpaired Student’s *t* test (**M**, **R**), Anova with Tukey’s correction (**N**, **P**), Anova with Dunnet’s test (**E**) or one-sided Wilcoxon test (**I**). *p < 0.05; **p < 0.01; ***p < 0.001.

In the randomized phase 2 H9H-MC-JBAJ study, we identified ANG as one of the most significant negative prognostic plasma proteins in patients receiving gemcitabine as a single agent. With an updated median follow-up of 28.85 (95% CI: 24.21–NA) months, patients with high vs. low ANG levels had mOS (95% CI) of 5.65 (3.32–8.84) vs 12.48 (7.69–24.94) months, respectively [HR (95% CI) high vs low= 2.78 (1.53–5.03); *p* = 0.0007] (Fig. 1A). Most importantly, inhibition of ALK5 with galunisertib reversed this resistance, and ANG emerged as a significant positive predictive marker for the combination of galunisertib plus gemcitabine. Indeed, ANG-high patients treated with galunisertib plus gemcitabine had a mOS (95% CI) of 8.90 (6.80–14.23) months compared to 5.65 (3.32–8.84) months in those treated with gemcitabine alone [HR (95% CI) galunisertib + gemcitabine vs gemcitabine + placebo=0.57 (0.35–0.92); *p* = 0.023] (Fig. 1A).

### Angiogenin functions as a negative prognostic factor in PDAC models by promoting a protumorigenic, M2-like phenotype in TAMs

To dissect the molecular and cellular mechanisms underlying the significant response of ANG-high PDAC patients to ALK5 inhibition, we first analyzed orthotopic models from six distinct murine PDAC cell lines. These cell lines were derived from tumors arising in either LSL-Kras^G12D/+^; p53^R172H/+^; PdxCre^tg/+^ (KPC: FC1245, FC1199, B6KPC, MET-610) or LSL-Kras^G12D/+^; PdxCre^tg/+^ (KC: DT4313, DT6606) mice. We observed significant differences in both tumor expression (Fig. 1B) and circulating plasma levels (Fig. 1C) of Ang in mice bearing orthotopic tumors from these different cell lines. Based on differences in mOS durations, we clustered the models into two groups: one with low or undetectable Ang expression and a long mOS duration (DT6606: 73 days; MET-610: 87.5 days; DT4313: 94 days), and a second, more aggressive group with high Ang expression and a significantly shorter mOS duration (FC1245: 20 days; FC1199: 26.5 days; B6KPC: 43 days) (Fig. 1D). Extensive immunophenotyping of these tumors (Figs. S1A, B and 1E, F) revealed a significant positive correlation between circulating Ang and the percentage of infiltrating TAMs (Fig. 1G) polarized to a M2 phenotype (R = 0.80705, *p* < 0.001) (Figs. 1H and S2A), while showing a negative correlation with the M1-TAMs population (R = –0.714074; *p* < 0.001) (Fig. S2B). Consistently with in vivo results, analysis of tumors from TCGA-PAAD database according to their annotation as primary tumors (*n* = 126) or metastasis (*n* = 22) revealed a positive correlation of ANG expression with the macrophage score from TIMER (*p* = 0.0007598, one-sided Wilcoxon test), as well as with the M2-TAMs proportion by CIBERSORT algorithm (*p* = 0.0493088, one-sided Wilcoxon test) in metastatic patients (Fig. 1I).

To further investigate the mechanisms underlying this correlation, we knocked down *Ang* in FC1245 cells by using two different short-hairpin RNAs (FC1245^shAng1^ and FC1245^shAng2^), or stably overexpressed *Ang* in DT4313 cells (DT4313^Ang^) (Fig. 1J). Upon orthotopic injection (Fig. 1K), mice bearing Ang-high FC1245^Scr^ or DT4313^Ang^ tumors exhibited significantly shorter mOS duration compared to those bearing FC1245^shAng^ or DT4313^NTC^ Ang-low respective controls. Specifically, mOS was 17 days for FC1245^Scr^ versus 24 days for FC1245^shAng^ [HR high/low= 3.5, 95% CI = 0.8975–13.65; *p* = 0.029], and 40 days for DT4313^Ang^ versus 128.5 days for DT4313^NTC^ [HR high/low= 3.488, 95% CI = 0.8956–13.59; *p* = 0.0092] (Fig. 1L). These results confirm the negative prognostic value of Ang expression in PDAC.

We next investigated the potential mechanistic role of Ang in promoting TAMs infiltration. Flow cytometry revealed a significantly higher (*p* < 0.05) infiltration of Cd45^+^/Cd11b^+^/F4/80^+^ TAMs in FC1245^Scr^ and DT4313^Ang^ tumors compared to their Ang-negative FC1245^shAng^ or DT4313^NTC^ respective counterparts (Fig. 1M). More importantly, Ang-high tumors had a significantly (*p* < 0.01) lower proportion of Cd86^+^/Cd204^-^ M1-polarized TAMs and a higher proportion of Cd86^-^/Cd204^+^ M2-polarized TAMs than did their respective Ang-low controls (Figs. 1N and S2C). Immunohistochemical analysis further confirmed the increased infiltration of Cd206^+^ M2-TAMs in Ang-expressing tumors (Fig. S2D–F), supporting the role of Ang in skewing macrophage polarization toward a pro-tumorigenic phenotype.

To assess the effect of the paracrine crosstalk between tumor-derived Ang and TAMs on their polarization, we employed co-culture models of bone marrow-derived macrophages (BMDM) with tumor cells modified for Ang expression. In this setup, a hydrophobic barrier physically separates the two cell types while allowing paracrine interactions (Fig. 1O). In this model, stimuli from recombinant Ang (rAng), as well as co-cultures with DT4313^Ang^-overexpressing cells, decreased the expression of M1 markers *Nos2* and *Cd86* and increased that of the M2 ones *Arg1* and *Mrc1* in BMDM if compared with BMDM left unstimulated, as a single culture or co-cultured with DT4313^NTC^ control cells (Fig. 1P). Consistently, genetic silencing of Ang in FC1245, as well as its depletion from the conditioned medium (CM) by immunoprecipitation, resulted in increased expression of M1 and decreased expression of M2 markers in BMDM (Figs. 1P and S3A–D). These findings were independently confirmed using an alternative co-culture system involving RAW264.7 murine macrophages and PDAC cells (Fig. S4A, B).

The M2 polarization state in TAMs has been associated with increased cellular elongation [16]. To investigate this morphological hallmark, we analyzed the actin cytoskeleton in BMDMs using phalloidin staining. As expected, TGFβ-polarized M2 BMDMs exhibited an elongated morphology with a well-defined leading and trailing edge, whereas IFNγ-polarized M1 BMDMs appeared more rounded and irregular in shape (Fig. S5A). Consistent with these findings, BMDMs stimulated with rAng displayed markedly increased cellular elongation compared to unstimulated naïve controls (Fig. S5A). qPCR analysis of M1 and M2 markers confirmed the polarization status of BMDM in this experimental setting (Fig. S5B).

To further characterize the immunosuppressive activity of these M2-polarized BMDM, we co-cultured activated T cells in a 1:5 ratio with BMDM unstimulated controls or pre-stimulated with rAng, and evaluated the ability of BMDM to interfere with IFNγ production from T cells (Fig. 1Q). We measured a significant reduction of IFNγ expression in the CD8^+^ T fraction, but not in the CD4^+^ one, when T cells were co-cultured with BMDM pre-conditioned with rAng compared to those left unstimulated (Fig. 1R), without any induction of T cell death (Fig. S6).

Collectively, these results indicate that ANG functions as a negative prognostic factor in PDAC and promotes a protumorigenic, M2-like phenotype in TAMs.

### EGFR is the functional receptor for Ang on TAMs and triggers autocrine Tgfβ activation

**Fig. 2:**
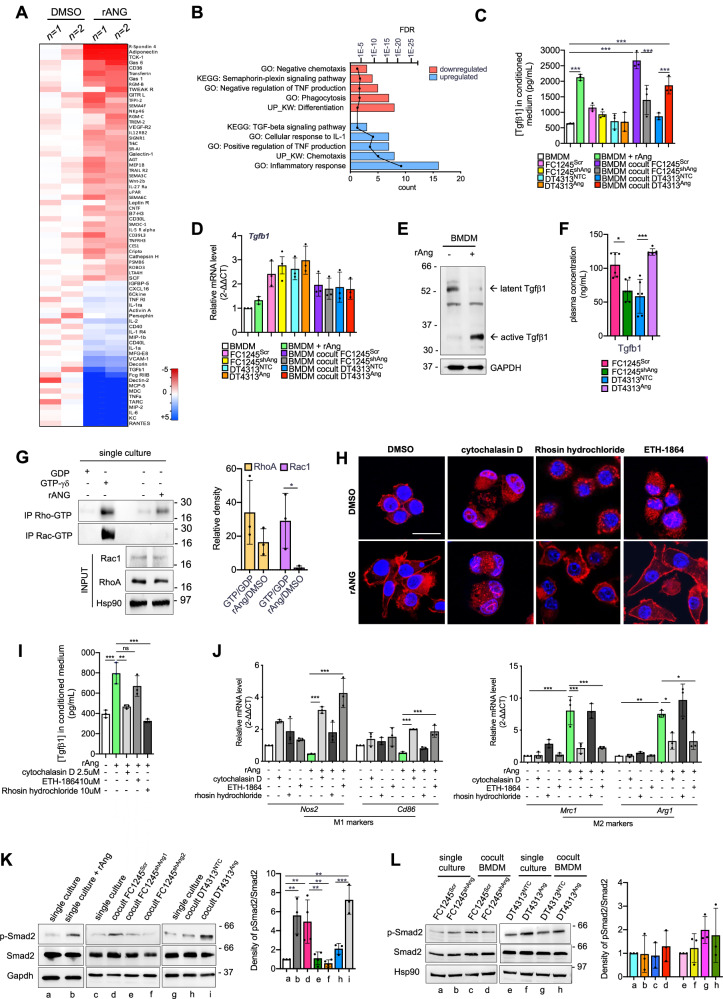
Angiogenin induces the release of Tgfβ from TAMs by promoting actin cytoskeleton contractility. Heat Map of hierarchically clustered proteins in BMDMs left unstimulated or stimulated with rAng for 48 h (**A**). Significantly upregulated (blue) and downregulated (red) Gene Ontology terms and enrichment *P* values by DAVID (functional annotation clustering) are shown in (**B**). **C** Tgfβ1 concentration in the supernatants of BMDMs, FC1245^Scr^, FC1245^shAng^, DT4313^NTC^ or DT4313^Ang^ as single cultures or in co-culture, measured by ELISA. Data are expressed as mean ± s.d. (*n* = 3). **D** qPCR of *Tgfb1* in BMDM, FC1245^Scr^, FC1245^shAng^, DT4313^NTC^ or DT4313^Ang^ as single cultures or in co-culture. Data are expressed as mean ± s.d. (*n* = 3). **E** Representative western blot of Tgfβ1 showing increased ratio of active to total (latent) Tgfβ1 in BMDMs, with a significant elevation of the active form in response to Ang exposure. Gapdh was used as a loading control. **F** Plasma concentration of Tgfβ1 in C57BL6/J mice bearing FC1245^Scr^, FC1245^shAng^, DT4313^NTC^ or DT4313^Ang^ tumors. Data are expressed as mean ± s.d. (*n* = 6). **G** Rho/Rac activation assay in BMDM treated with DMSO or rAng for 48 h. The endogenous active GTP-bound form of RhoA and Rac1 was pulled down and detected by Western blotting. Input was 5%. Densitometric analysis from three independent experiments is shown as GTP/GDP or rAng/DMSO ratios. **H** Representative immunofluorescence images of BMDM stimulated with rAng or left unstimulated, and treated with 2.5 μM cytochalasin D, 15 µM ETH-1864, 10 μM rhosin hydrochloride or DMSO for 48 h. BMDMs were stained with Phalloidin and counterstained with DAPI. Scale bar = 20 μm. **I** Tgfβ1 concentration in the supernatants of BMDM unstimulated or stimulated with rAng and treated as indicated for 48 h, measured by ELISA. Data are expressed as mean ± s.d. (*n* = 3). **J** qPCR analysis of M1 (*Nos2*, *Cd86*) and M2 (*Arg1*, *Mrc1*) markers in BMDM left unstimulated or stimulated with rAng, and treated as indicated for 48 h. Data are shown as mean ± s.d (*n* = 3). **K** Representative western blot of pSmad2 and Smad2 in BMDMs unstimulated or stimulated with rAng, and as single cultures or in co-culture with FC1245 or DT4313 transduced as indicated. Gapdh was used as a loading control. Densitometry results from three independent experiments are shown. **L** Representative western blot of pSmad2 and Smad2 in FC1245 or DT4313 transduced as indicated as single cultures or in co-culture with BMDMs. Hsp90 was used as a loading control. Densitometry results from three independent experiments are shown. *P* values were calculated using two-tailed unpaired Student’s *t* test (**F**, **G**) or ANOVA and Tukey’s test (**C**, **D**, **I**, **J**, **K**, **L**). *p < 0.05; **p < 0.01; ***p < 0.001.

To investigate the effects of Ang on TAM-secreted factors, we profiled the conditioned medium of BMDMs upon stimulation with rAng using a multi-analyte panel targeting 400 cytokines, chemokines, growth factors, and related proteins. Compared to unstimulated controls, rAng stimulation resulted in 71 differentially secreted proteins from BMDMs (*p* < 0.05). Gene ontology analysis using the DAVID functional annotation tool revealed a significant downregulation of M1-associated signatures and biological processes such as phagocytosis. In contrast, there was a marked enrichment in M2-associated processes, including inflammatory responses and TGFβ signaling (Fig. 2A, B).

We then used co-culture models to measure the activation of TGFβ signaling in BMDMs and PDAC cells modified for Ang expression, either cultured alone or in combination. BMDMs stimulated with rAng or co-cultured with Ang-high FC1245^Scr^ or DT4313^Ang^ cells secreted significantly higher levels of TGFβ1 (*p* < 0.001) compared to unstimulated BMDMs or those co-cultured with Ang-low controls (FC1245^shAng^ or DT4313^NTC^ controls) (Fig. 2C). Notably, this increase was observed at the protein level, while *Tgfb1* mRNA levels remained unchanged (Fig. 2D), suggesting a post-transcriptional regulation. Consistent with these findings, rAng stimulation increased the ratio of active to total (latent) Tgfβ1 in BMDM, with a significant elevation of the active form in response to Ang exposure (Fig. 2E). Consistent with our in vitro findings, we observed significantly elevated circulating levels of plasma Tgfβ1 in Ang-high orthotopic models (Fig. 2F).

It is well established that actin cytoskeleton dynamics facilitate the release and activation of Tgfβ from its latent complex [17]. The Rho family GTPases RhoA and Rac1 are the most studied regulators of actin cytoskeletal organization [18]. Thus, we evaluated the role of Ang on the activation of these GTPases. By specifically pulling down the active GTP-bound form of Rho and Rac1 GTPases, we found active RhoA in BMDM upon stimulation with rAng (Fig. 2G). To dissect the functional relevance of these cytoskeletal regulators, we pharmacologically inhibited actin and actin-associated signaling pathways by using either cytochalasin D to inhibit actin polymerization, rhosin hydrochloride to inhibit RhoA-GTPase dynamics, and EHT-1864 to inhibit Rac1-GTPase activity as a negative control. Treatment of BMDMs with cytochalasin D or rhosin hydrochloride, but not with EHT-1864, significantly reversed the Ang-induced morphological changes, as shown by phalloidin staining (Fig. 2H). More importantly, both cytochalasin D and rhosin hydrochloride nearly abolished Tgfβ1 secretion (Fig. 2I) and the M2 polarization of BMDM (Fig. 2J). These findings suggest that RhoA is required for the Ang-driven release of active Tgfβ1 and the subsequent M2 polarization of TAMs. In co-culture experiments, the increased Tgfβ secretion upon stimuli from tumor Ang significantly enhanced Smad2 phosphorylation in macrophages (Figs. 2K and S4C), but not in PDAC cells, either as single cultures or in co-culture with them (Fig. 2L). These results indicate that Ang promotes cytoskeleton contractility in TAMs, resulting in the release of active Tgfβ1 and the autocrine activation of the ALK5 signaling pathway.

**Fig. 3:**
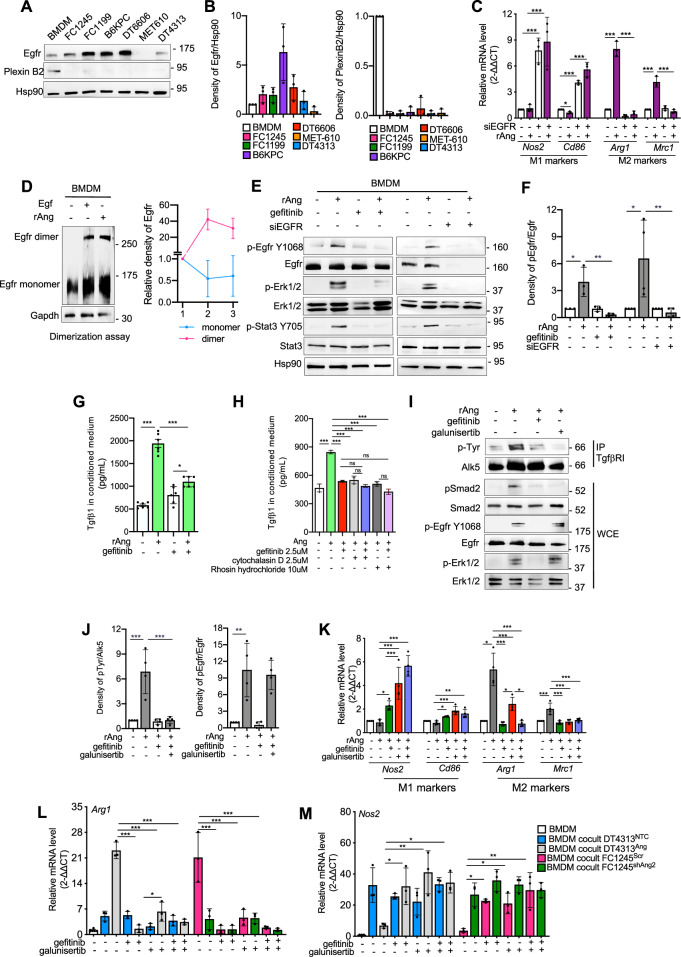
EGFR is the functional receptor for Ang on TAMs and triggers autocrine Tgfβ activation. **A** Representative western blot of Egfr and Plexin B2 in whole cell extracts of BMDM, FC1245, FC1199, B6KPC, DT6606, MET-610 and DT4313 cell lines. Hsp90 was used as a loading control. **B** Densitometry of Egfr and PlexinB2 relative to 3 independent replicates. **C** qPCR analysis of *Nos2*, *Cd86, Arg1 and* *Mrc1* in BMDMs transiently transfected with siEgfr or siRNA control vector, left unstimulated or stimulated with rAng for 48 h. Data are shown as mean ± s.d (*n* = 3). **D** Dimerization of Egfr in BMDMs stimulated with Egf (400 ng/ml) or Ang (1μg/ml) for 1 h and crosslinked with BS3. Both the Egfr monomer and dimer are visible in the immunoblot. 500μg of lysates were used. Gapdh served as a loading control. Densitometric quantification of monomeric and dimeric fractions from 3 independent experiments is shown. **E** Representative western blot of p-Egfr Y1068, Egfr, p-Erk1/2, Erk1/2, p-Stat3 Y705, and Stat3 in BMDMs stimulated with rAng or left unstimulated, treated with 2.5 μM gefitinib or DMSO for 48 h. HSP90 was used as a loading control. **F** Densitometry measurements of pEGFR protein bands relative to total Egfr from at least 3 independent experiments, as shown in (**E**). **G** Tgfβ1 concentration in the supernatants of BMDMs treated as indicated for 48 h, measured by ELISA. Data are expressed as mean ± s.d. (*n* = 6). **H** Tgfβ1 concentration in the supernatants of BMDM stimulated with rAng and treated with 2.5 μM gefitinib, 2.5 μM cytochalasin D or 10 μM rhosin hydrochloride and their combinations for 48 h. Data are expressed as mean ± s.d. (*n* = 3). **I** Representative western blot of phospho-Tyrosine (p-Tyr) after immunoprecipitation with Tgfβr1 in BMDM stimulated with rAng and treated with 2.5 μM gefitinib, 5 μM galunisertib, their combination or DMSO for 48 h, and of p-Smad2, Smad2, p-Egfr Y1068, Egfr, p-Erk1/2 and Erk1/2 from WCE. **J** Densitometry measurements of pTyr or pEgfr protein bands relative to total TgfβRI or Egfr, respectively, from 4 independent experiments as shown in (**I**). **K** qPCR analysis of M1 (*Nos2*, *Cd86*) and M2 (*Arg1*, *Mrc1)* markers in BMDM unstimulated or stimulated with rAng and treated as indicated for 48 h. Data are expressed as mean ± s.d. (*n* = 4). qPCR analysis of *Arg1* (**L**) and *Nos2* (**M**) markers in BMDMs as single cultures or co-cultured with FC1245^Scr^, FC1245^shAng^, DT4313^NTC^ or DT4313^Ang^ and treated with gefitinib, galunisertib, their combination or DMSO for 48 h. Data are expressed as mean ± s.d. (*n* = 3). *P* values in **C**, **F**, **G**, **H**, **J**, **K**, **L**, **M** were calculated using ANOVA and Tukey’s test. *p < 0.05; **p < 0.01; ***p < 0.001.

To identify the receptor mediating Ang signaling in TAMs, we first analyzed the expression of Egfr and PlexinB2 in a panel of murine PDAC cells and in BMDM. While Egfr was variably expressed in both PDAC cells and macrophages, Plexin B2 expression was predominantly restricted to macrophages (Figs. 3A, B and S7A). Genetic inhibition of Egfr, as well as its pharmacological blockade using the selective tyrosine kinase inhibitor gefitinib, almost completely abrogated the M2 polarization of BMDM induced by rAng (Figs. 3C and S7B). In contrast, knockdown of Plexin B2 did not affect Ang-driven polarization (Fig. S7C), suggesting that Egfr, rather than Plexin B2, functions as the primary receptor for Ang in this context.

To further investigate whether Egfr functions as the receptor for Ang on macrophages, we stimulated serum-starved BMDMs with rAng or Egf as a positive control and assessed Egfr dimerization. In BMDMs, Egfr predominantly existed in its monomeric form. However, stimulation with rAng induced a partial Egfr dimerization, almost comparable to that induced by Egf (Fig. 3D). This led to an increased Egfr phosphorylation at Tyr 1068, inducing the activation of the downstream mediators Stat3 and Erk1/2 (Figs. 3E, F and S7D). Notably, this effect was completely reversed by both genetic and pharmacological inhibition of Egfr (Figs. 3E, F and S7D). Together, these findings support the finding that Ang functions as a ligand for EGFR in macrophages, promoting its dimerization and downstream activation.

We next investigated the mechanisms by which the Ang/Egfr axis regulates Tgfβ1 secretion in macrophages. Treatment with gefitinib completely suppressed Tgfβ1 secretion induced by rAng (Fig. 3G). Moreover, combining gefitinib with rhosin hydrochloride did not produce any additive inhibitory effect (Fig. 3H), further confirming that cytoskeleton dynamics play a central role in mediating Tgfβ1 secretion following Ang-induced EGFR activation.

We then investigated the role of the Egfr/Tgfβ axis in sustaining the M2 polarization of TAMs in response to Ang stimulation. Inhibition of Egfr with gefitinib impaired Alk5 activation and subsequent Smad2 phosphorylation in rAng-stimulated BMDMs, while inhibition of Alk5 with galunisertib did not impact Egfr activation (Figs. 3I, J and S7E), indicating that Egfr signaling acts upstream of Tgfβ signaling in this context. qPCR analysis of BMDMs revealed a comparable shift from M2 to M1 phenotype following inhibition of either Egfr or Alk5 under stimuli from rAng (Fig. 3K), as well as in co-cultures with Ang-high FC1245^Scr^ or DT4313^Ang^ cells (Fig. 3L, M). This effect was not observed in unstimulated BMDM, nor in BMDM co-cultured with the Ang-low FC1245^shAng^ or DT4313^NTC^ controls (Fig. 3K–M). Notably, combined inhibition of either Egfr or Alk5 did not produce any additive effect, further supporting a model in which Egfr activation prompts Alk5 signaling to promote M2-like polarization of macrophages in response to Ang.

Collectively, our data indicate that EGFR acts as a functional receptor for Ang on TAMs, promoting RhoA-mediated cytoskeletal contractility that leads to the release of active Tgfβ1. This, in turn, triggers an autocrine activation of ALK5 signaling in macrophages, sustaining their M2 polarization in response to Ang stimulation.

### The Ang/Tgfβ signaling axis in TAMs sustains chemoresistance via paracrine Tnfα in PDAC cells

To investigate the role of the Ang/Egfr/Tgfβ signaling axis in mediating the response of Ang-high tumors to combined ALK5 inhibition and chemotherapy, we treated PDAC cells with increasing doses of gemcitabine in different culture medium conditions. When PDAC cells were cultured in normal medium, no differences in gemcitabine EC50 values were observed between Ang-high (FC1245^Scr^, DT4313^Ang^) and Ang-low (FC1245^shAng^, DT4313^NTC^) cells upon galunisertib (Fig. S8A). However, when treatment occurred with the CM from co-cultures with BMDM, Ang-high cells exhibited significantly higher EC50 values compared to their Ang-low counterparts (Fig. S8B), suggesting that TAMs-derived factors contribute to Ang-mediated chemoresistance. Most importantly, pretreatment of co-cultures with the ALK5 inhibitor galunisertib significantly reduced this resistance (Fig. S8B), implicating TGFβ signaling in TAMs as a key mediator. We ruled out a direct effect of TGFβ on PDAC cells by treating them in the CM from untreated co-cultures and observing no changes in gemcitabine sensitivity upon addition of galunisertib (Fig. S8C). These results support the finding that tumor-derived Ang induces TGFβ signaling in TAMs, which in turn promotes chemoresistance through additional paracrine factors beyond TGFβ itself.

**Fig. 4:**
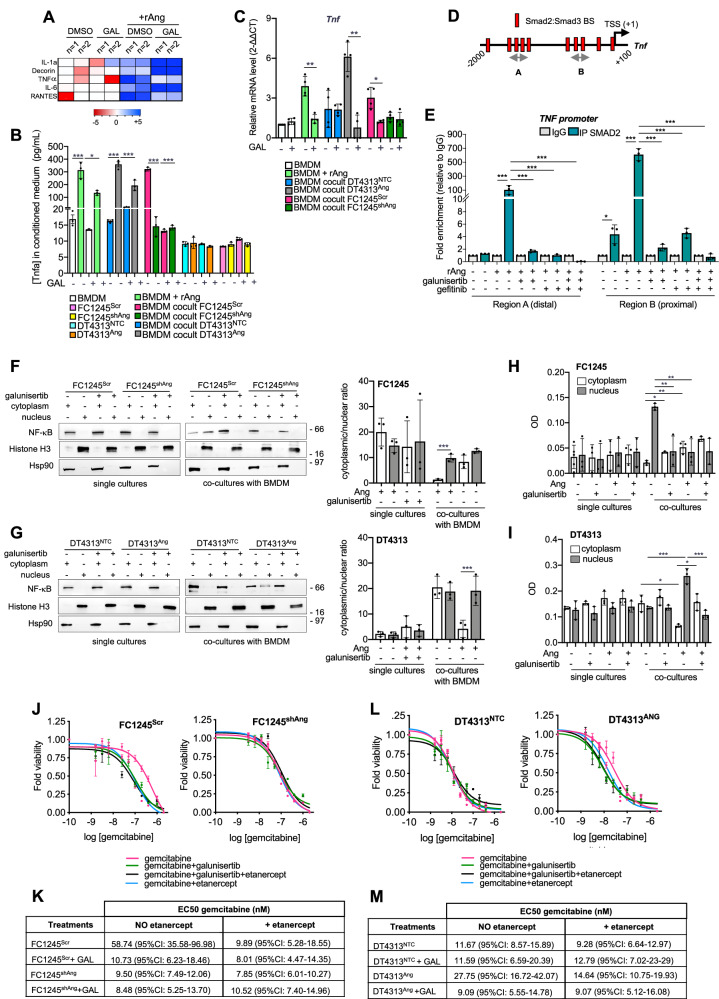
TAMs-derived Tnfα acts as a mediator of TGFβ signaling in sustaining chemoresistance of Ang-high PDAC cells. **A** Heat map of the most significantly regulated cytokines from supernatants collected from BMDM unstimulated or stimulated with rAng and treated with 5 μM galunisertib for 48 h. Cytokine nomenclature is shown. **B** Tnf-α concentration in the supernatants of BMDMs, FC1245^Scr^, FC1245^shAng^, DT4313^NTC^ or DT4313^Ang^ as single cultures or in co-culture and treated for 48 h with 2.5 μM galunisertib, measured by ELISA. Data are expressed as mean ± s.d. (*n* = 3). **C** qPCR of *Tnf* in BMDMs unstimulated or rAng-stimulated, either as a single culture or in coculture with FC1245^Scr^, FC1245^shAng^, DT4313^NTC^ or DT4313^Ang^ and treated for 48 h with 2.5 μM galunisertib. Data are expressed as mean ± s.d. (*n* = 4). **D** Schematic representation of Tnf promoter (available at UCSC Genome Browser GRCm38/mm10) with the position of ChIP probes (gray double arrowhead) and consensus Smad-binding sites (vertical slashes) relative to the transcription starting site (TSS). **E** ChIP-qPCR of Smad2 occupancy at the Tnf promoter in RAW264.7 macrophages stimulated with rAng, in the presence or absence of 2.5 μM gefitinib, 5 μM galunisertib or their combinations. The y-axis represents relative promoter enrichment, normalized on input material. IgG was set to 1. Data are represented as mean ± s.d. (*n* = 3). *P* values in (**B**, **C**, **E**) were calculated using ANOVA and Tukey’s test. *p < 0.05; **p < 0.01; ***p < 0.001. **F**, **G** Representative Western blots of Nf-κB cytoplasmic and nuclear fractions in FC1245^Scr^ and FC1245^shAng^ (**F**) or in DT4313^NTC^ and DT4313^Ang^ (**G**) as single cultures or in co-culture with BMDMs, treated for 48 h as indicated. Hsp90 and histone H3 were used as cytoplasmic or nuclear markers, respectively. The cytoplasmic/nuclear ratio of Nf-κB from 3 independent experiments is shown. **H**, **I** Nf-κB p65 DNA binding ELISA in cytoplasmic or nuclear extracts from FC1245 (**H**) or DT4313 (**I**) cells transduced and treated as indicated, either as single cultures or co-cultured with BMDM (OD = 450 nm). **J**–**M** Cell viability of FC1245 (**J**, **K**) or DT4313 (**L**, **M**) co-cultured with BMDMs and transduced as indicated, after 72 h of treatment with gemcitabine alone or in combination with 2 μM galunisertib, in the presence or absence of 5μg/mL Etanercept. GAL: galunisertib. Tables in **K**, **M** indicate EC50 values and 95% CIs of gemcitabine. *P* values were calculated using ANOVA and Tukey’s test (**B**, **E**, **F**, **G**, **H**, **I**) or paired Student’s *t* test (**C**). *p < 0.05; **p < 0.01; ***p < 0.001.

To identify those putative paracrine mediators, we profiled the levels of differentially secreted proteins in the CM from BMDM cultures in basal conditions or upon stimuli from rAng, in the presence or absence of galunisertib. Among the most significantly upregulated factors was Tumor Necrosis Factor-alpha (Tnfα), whose secretion was induced by rAng dependently on ALK5, as galunisertib completely abolished its release (Fig. 4A). In support of this, ELISA analysis of CM from PDAC cells and BMDMs, either alone or in co-culture, revealed a significant increase in secreted Tnfα exclusively in co-cultures involving Ang-high FC1245^Scr^ or DT4313^Ang^ cells, which was fully suppressed by treatment with galunisertib (Fig. 4B). qPCR analysis confirmed that macrophages, rather than tumor cells, were responsible for the secretion of Tnfα induced by the Ang/Tgfβ axis (Figs. 4C and S9A). Chromatin immunoprecipitation (ChIP) of Smad2 using different sets of primers spanning the putative binding sites within the *Tnf* promoter revealed Ang-dependent Smad2 enrichment at both distal and proximal *Tnf* promoter regions, with a more than 100-fold increase in *Tnf* signal over non-specific IgG in macrophages stimulated with rAng (*p* < 0.001) (Fig. 4D, E). Notably, treatment with either galunisertib or gefitinib as single agents suppressed this transcriptional activation of *Tnf* induced by Ang, with no additive effect observed upon their combination (Fig. 4E).

TNFα is a well-established regulator of non-canonical NF-κB signaling [19, 20], a pathway known to contribute to treatment resistance in PDAC [19]. Moreover, recent evidence has linked TNFα to the transcriptional signature of M2-polarized TAMs [21], and its overexpression has been associated with poor prognosis in PDAC [22]. Based on these findings, we next investigated whether the TGFβ/TNFα axis in macrophages could sustain the paracrine activation of Nf-κb in Ang-high PDAC cells, thereby promoting chemoresistance. We observed increased nuclear translocation of NF-κB in Ang-high FC1245^Scr^ or DT4313^Ang^ cells co-cultured with BMDM compared to single cultures, but not in co-cultures of Ang-low FC1245^shAng^ and DT4313^NTC^ controls (Fig. 4F, G). This was paralleled by a consistent increase in NF-κB activation (Figs. 4H, I and S9B). Notably, NF-κB nuclear translocation and activation were completely abrogated by galunisertib treatment (Figs. 4F–I and S9B), underscoring the central role of TGFβ-dependent TNFα signaling in mediating this response.

To then determine the role of macrophage-derived Tnfα in modulating chemoresistance of Ang-high PDAC cells, we used the CM from established co-cultures pre-treated or not with galunisertib to treat PDAC cell lines with increasing concentrations of gemcitabine in combination with the TNF inhibitor etanercept (Fig. 4J–M). We observed a comparable modulation of the response to gemcitabine achieved by either Tnfα or Alk5 inhibition in FC1245^Scr^ or DT4313^Ang^, but not in FC1245^shAng^ or DT4313^NTC^, respectively, and their combination did not obtain additive effects, indicating a serial activation of these two pathways rather than a parallel and synergistic effect in sustaining chemoresistance of Ang-high cells. Likewise, stimulation of FC1245^Scr^ or DT4313^Ang^ cells with rTnfα (Fig. S9C) significantly increased the EC50 values of gemcitabine in Ang-low FC1245^shAng^ and DT4313^NTC^ cells, but had no additional effects on Ang-high FC1245^Scr^ or DT4313^Ang^ cells (Fig. S9D–G), consistent with the role of macrophage-derived Tnfα as a key mediator of chemoresistance in the Ang-high setting. Most importantly, rTnfα almost completely abrogated the efficacy of galunisertib in increasing the sensitivity of Ang-high PDAC cells to gemcitabine (Fig. S9D–G). These results indicate that the activation of the TGFβ signaling axis in macrophages reduces the efficacy of gemcitabine in Ang-high tumors by promoting a TNFα-driven paracrine mechanism of resistance, likely relying on Nf-κb activation.

**Fig. 5:**
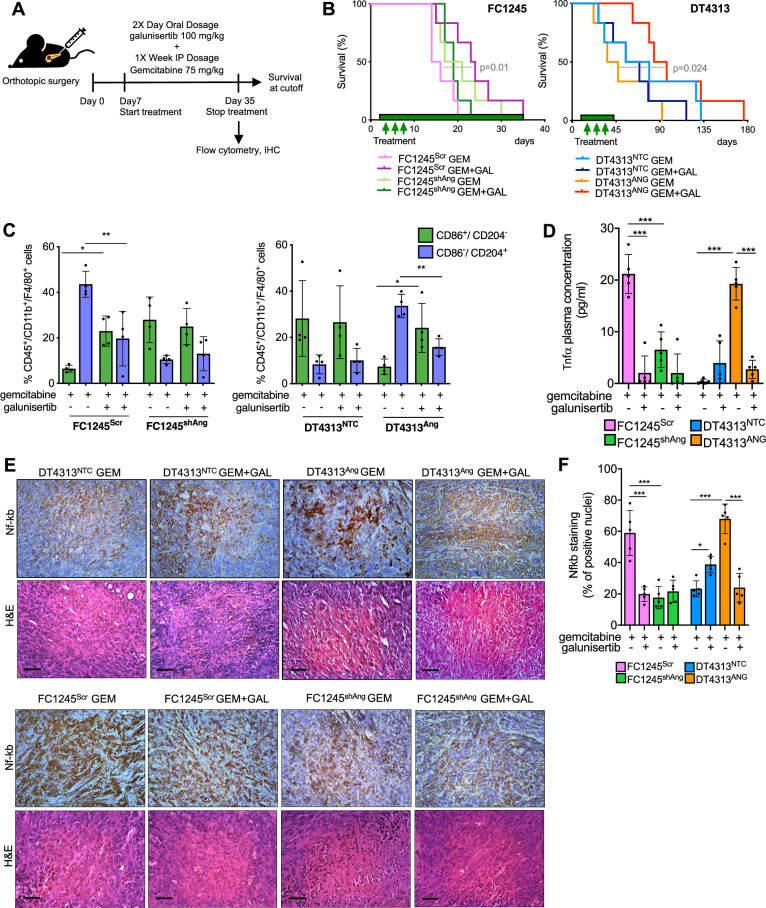
Inhibition of TGFβ signaling improves the activity of gemcitabine in Ang-high PDAC tumors. **A** Schematic illustration of the treatment schedule. **B** mOS of mice bearing FC1245^Scr^ vs FC1245^shAng^ or DT4313^NTC^ vs DT4313^Ang^ orthotopic tumors treated with gemcitabine, galunisertib, their double combinations or their respective vehicles as control (*n* = 6). **C** Flow cytometry analysis M2 (Cd45^+^/Cd11b^+^/F4/80^+^/Cd204^+^/Cd86^-^)/M1(Cd45^+^/Cd11b^+^/F4/80^+^/Cd204^-^/Cd86^+^) ratio of TAMs in tumors from mice bearing FC1245 or DT4313 cells transduced as indicated and treated for 4 weeks with gemcitabine, galunisertib, or their double combinations (*n* = 4). **D** Plasma levels of Tnf-α measured by ELISA in FC1245^Scr^ vs FC1245^shAng^ or DT4313^NTC^ vs DT4313^Ang^ orthotopic tumors treated as indicated (*n* = 5). **E** Representative images for DT4313 or FC1245 tumors stained with Nf-κB and H&E. Scale bar=60 μm. **F** Quantification of paraffin sections from orthotopic murine PDAC tumors stained as indicated in (**E**). Data are expressed as mean ± s.d. (*n* = 5). *P* values in (**C**, **D**, **F**) were calculated by one-way ANOVA and Tukey’s test. *p < 0.05; **p < 0.01; ***p < 0.001.

To validate the therapeutic relevance of the Ang/TGFβ axis in vivo, we evaluated the efficacy of combined ALK5 inhibition and chemotherapy in orthotopic models. Mice bearing Ang-high (FC1245^Scr^ and DT4313^Ang^) or Ang-low (FC1245^shAng^ and DT4313^NTC^) tumors were randomized to receive galunisertib, gemcitabine, both agents in combination, or their respective vehicles (Fig. 5A). In Ang-high tumors, the combination of gemcitabine plus galunisertib significantly prolonged mOS compared to gemcitabine alone (FC1245^Scr^: 15 vs 23.5 days, HR = 3.244, 95% CI = 0.855–12.31, *p* = 0.01; DT4313^Ang^: 41.5 vs 90.5 days, HR = 3.055, 95% CI = 0.822–11.35, *p* = 0.024). In contrast, no survival benefit was observed in Ang-low tumors treated with the combination (FC1245^shAng^: 19 vs 19 days, HR = 0.7074, 95% CI = 0.2243–2.231, *p* = 0.4998; DT4313^NTC^: 67 vs 62 days, HR = 0.6083, 95% CI = 0.1894–1.954, *p* = 0.3332) (Fig. 5B).

Angio-high tumors treated with the combination of gemcitabine plus galunisertib also exhibited a significantly reduced infiltration of CD86^-^/CD204⁺ M2-like TAMs (*p* < 0.001), which was paralleled by increased infiltration of CD86⁺/CD204⁻ M1-like TAMs (*p* < 0.05), compared to tumors treated with gemcitabine alone (Figs. 5C and S10). Most importantly, high levels of circulating Tnfα were observed only in mice bearing Ang-high tumors treated with gemcitabine, while co-treatment with galunisertib almost completely abrogated Tnfα secretion in the plasma of these mice (Fig. 5D).

Consistent with our in vitro findings, Ang-high tumors from mice treated with gemcitabine exhibited moderate nuclear staining of Nf-κb, whereas co-treatment with galunisertib significantly reduced Nf-κb nuclear localization, which was instead predominantly observed in the cytoplasm of tumor cells (Fig. 5E, F). In contrast, Nf-κb expression was absent in the nuclei of Ang-low FC1245^shAng^ and DT4313^NTC^ tumor cells from mice treated with gemcitabine alone. Interestingly, combination therapy in DT4313^NTC^ tumors led to a paradoxical nuclear translocation of NF-κB (Fig. 5E, F), potentially reflecting adaptive resistance mechanisms to TGFβ inhibition, as recently described in association with the autotaxin axis [23]. These results indicate that pharmacological blockade of ALK5 signaling in TAMs suppresses their M2 polarization and Tnfα secretion, thereby preventing the paracrine Nf-κb activation in PDAC cells that underlies the resistance of Ang-high tumors to the cytotoxic effects of chemotherapy.

Collectively, these findings support a model in which the Ang/EGFR/TGFβ axis confers chemoresistance to PDAC cells through a paracrine mechanism. Specifically, TGFβ autocrine signaling in macrophages leads to the Smad2-dependent transcriptional induction of Tnfα, whose secretion activates non-canonical NF-κB signaling in neighboring tumor cells, ultimately resulting in resistance to gemcitabine.

### Plasma TNFα levels are reduced by galunisertib plus gemcitabine and predict treatment response in ANG-high PDAC patients

**Fig. 6:**
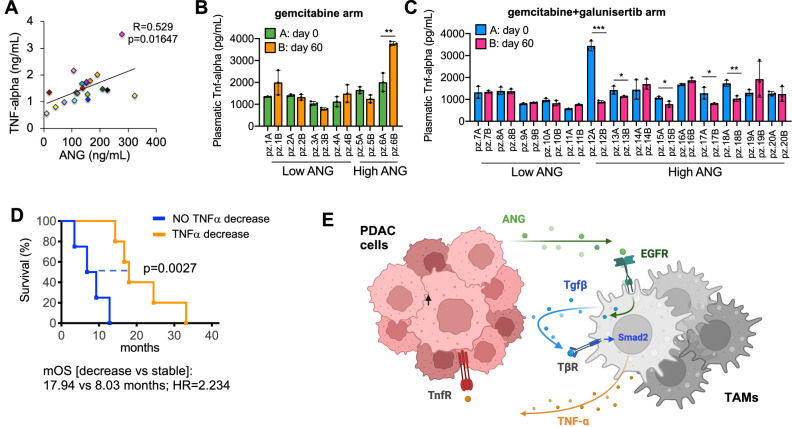
Plasma levels of TNFα are decreased by galunisertib plus chemotherapy and predict response to this treatment strategy in patients with ANG-high PDAC. **A** Pearson’s correlation between plasma levels of ANG and TNFα at baseline in 20 patients enrolled in the randomized phase II H9H-MC-JBAJ trial (ANG-high *n* = 12; ANG-low *n* = 8). **B**, **C** Plasma levels of TNFα are decreased by galunisertib plus chemotherapy and predict response to this treatment strategy in patients with ANG-high PDAC. Plasmatic TNFα was measured at baseline (day 0) and after two cycles of treatment (day 60) in patients within the H9H-MC-JBAJ trial treated with placebo plus gemcitabine as the control arm (B) or with galunisertib plus gemcitabine as the experimental arm (**C**). Data are expressed as mean ± s.d. *P* values in (**B**, **C**) were calculated by paired Student’s *t* test. *p < 0.05; **p < 0.01; ***p < 0.001. **D** mOS of ANG-high patients allocated to galunisertib plus gemcitabine, which had a significant decrease in plasmatic TNFα (orange line) versus those with no decrease in plasmatic TNFα (blue line). mOS [decrease vs stable]: 17.94 vs 8.03 months; HR = 2.234; *p* = 0.0027. **E** Graphical abstract of proposed mechanism.

We validated the clinical relevance of the ANG/TGFβ/TNFα axis as a mechanism of chemoresistance in PDAC using samples from patients enrolled at our institution in the randomized phase II H9H-MC-JBAJ trial. As an initial step, we observed a significant positive correlation between baseline circulating levels of ANG and TNFα (Pearson’s R = 0.529; *p* = 0.01647) (Fig. 6A).

We then analyzed TNFα plasma levels at baseline and after 60 days of treatment in patients stratified by ANG expression (above or below the median) and treatment arm (gemcitabine plus placebo vs gemcitabine plus galunisertib). In the control arm (gemcitabine plus placebo), no significant changes in TNFα levels were detected in either ANG-low or ANG-high patients (Fig. 6B).

In contrast, a significant reduction in circulating TNFα was observed in a subset of ANG-high patients treated with gemcitabine plus galunisertib (Fig. 6C). Importantly, this reduction in TNFα levels was associated with a significant improvement in median overall survival among ANG-high patients: those with decreased TNFα levels after treatment had a mOS of 17.94 months compared to 8.03 months in those with stable TNFα levels (HR = 2.234; *p* = 0.0027) (Fig. 6D). These findings strongly support the translational relevance of our preclinical models, indicating that elevated ANG levels in PDAC patients are associated with increased systemic TNFα, and may serve as a biomarker for identifying tumors driven by the ANG/TGFβ/TNFα axis and potentially responsive to TGFβ-targeted therapies.

## Discussion

PDAC remains one of the deadliest malignancies, largely due to its complex tumor microenvironment, intrinsic resistance to chemotherapy, and paucity of predictive biomarkers. In this study, we identify ANG as a clinically relevant predictive biomarker for response to the inhibition of the ALK5 signal pathway in patients with metastatic PDAC. Furthermore, we delineate the molecular and cellular mechanisms underlying this response, uncovering a critical role for ANG in mediating immune-driven chemoresistance. Our findings establish ANG as a novel regulator of TAMs polarization in PDAC. We demonstrate that ANG exerts its effects on TAMs by binding to and activating EGFR, triggering a RhoA-mediated cytoskeletal remodeling, and the subsequent release of active TGFβ1. This autocrine TGFβ signaling in TAMs sustains their M2 polarization and establishes a feed-forward loop that confers chemoresistance to adjacent tumor cells in a paracrine manner. Specifically, this resistance is mediated through the paracrine secretion of TNFα, which we identify as a transcriptional target of Smad2 in macrophages. TNFα activates the non-canonical NF-κB pathway in neighboring tumor cells, a pathway previously implicated in chemoresistance and poor prognosis in PDAC (Fig. 6E). Most importantly, our findings have an immediate clinical relevance by demonstrating that the pharmacological blockade of ALK5 signaling leads to reduced plasma levels of TNFα and potentiates response to chemotherapy in ANG-high PDAC patients. These results support ANG as both a mechanistic driver of TAMs-mediated chemoresistance and a predictive biomarker for stratifying patients who may benefit from combined ALK5 inhibition and chemotherapy.

TAMs represent the most abundant immune cell population within the immunosuppressive tumor microenvironment characteristic of PDAC. While extensive evidence supports the interaction between TAMs and PDAC cells in promoting several key hallmarks of cancer, including resistance to therapy [24], the specific contribution of EGFR signaling within this crosstalk has remained poorly understood. A limited number of studies have demonstrated that EGFR is expressed in human macrophages, where it regulates cytokine production and shapes macrophage responses, particularly in inflammatory contexts [25, 26]. In hepatocellular carcinoma (HCC), EGFR expression has been detected in liver-resident macrophages (Kupffer cells) in both human samples and murine models. Macrophage-specific deletion of EGFR in mice impairs hepatocarcinogenesis, and in human HCC, the presence of EGFR-positive macrophages is associated with poor patient survival, highlighting the clinical relevance of this signaling axis [27]. Despite this emerging evidence, the role of EGFR signaling in TAMs and the downstream molecular mechanisms that support chemoresistance, particularly in PDAC, remained largely unexplored. In the present study, we identify a previously unrecognized function for the EGFR ligand ANG in promoting TAMs polarization. We demonstrate that ANG engages EGFR on TAMs, initiating an autocrine activation of ALK5 signaling pathways that sustains chemoresistance in PDAC cells via paracrine Tnfα. These findings uncover a novel mechanism by which EGFR signaling, triggered by a non-canonical ligand, contributes to the tumor-supportive functions of TAMs. Importantly, our results establish EGFR as a key modulator of stromal cell behavior in PDAC, extending its known oncogenic roles beyond tumor epithelial cells to the broader tumor microenvironment.

The role of TNF-α-producing macrophages is emerging as increasingly significant in PDAC. Comparative analyses of classical and basal-like PDAC subtypes have revealed an enrichment of TNF-α-producing macrophages in the poorly differentiated basal-like subtype, which is associated with worse prognosis. Mechanistically, BRD4-driven activation of cJUN/AP1 promotes CCL2 expression in neoplastic cells, facilitating the recruitment of TNF-α–producing macrophages. In turn, TNF-α reprograms classical PDAC cells into a more aggressive, basal-like, and mesenchymal phenotype [21]. More recently, TNF-α–secreting macrophages have also been implicated in modulating the response to immune checkpoint inhibitors. These macrophages were confirmed as the primary source of TNF-α responsible for suppressing IL-33 expression in PDAC cells. In genetically engineered mouse models, the genetic deletion or pharmacological inhibition of Ccr2 reduced monocyte recruitment, resulting in markedly lower TNF-α levels, increased IL-33 expression, a reduced metastatic burden, and improved survival in the context of checkpoint blockade therapy [28]. In our study, we demonstrated that TNF-α is significantly regulated in macrophages by the Ang/Tgfβ axis, and that its paracrine activity promotes chemoresistance in PDAC cells through activation of the Nf-κb pathway. Most importantly, we validated these preclinical findings in a unique clinical setting of patients enrolled in a randomized clinical trial and treated with either an ALK5 inhibitor or placebo. We demonstrated that high ANG levels in PDAC patients are associated with elevated systemic TNF, that ALK5 inhibition leads to a significant reduction in circulating TNF in ANG-high patients, and that this decrease in TNF levels correlates with a significant improvement in overall survival.

We recently established chemokine (C-C motif) ligand 3 (CCL3) as a distinct positive predictive biomarker for TGFβ signaling inhibition in PDAC patients. Mechanistically, we demonstrated that tumor-derived CCL3 activates Tgfβ signaling in macrophages, inducing the secretion of the proinflammatory cytokine Lif, which in turn sustains a mesenchymal/basal-like phenotype in tumor cells. Inhibition of TGFβ signaling reduced Lif production and promoted a shift toward a more epithelial/classical phenotype in PDAC cells, thereby enhancing their sensitivity to chemotherapy [29]. In light of these findings, we investigated the potential interplay between CCL3 and ANG as predictive biomarkers of response to TGFβ inhibition. However, we found no correlation between baseline plasma levels of CCL3 and ANG in PDAC patients, and modulation of CCL3 expression in in vitro co-culture models did not affect ANG levels (data not shown). Nonetheless, the fact that these two independent mechanisms converge on the modulation of TGFβ signaling in macrophages reinforces the notion that the therapeutic benefit of ALK5 inhibition in PDAC may be primarily mediated through its effects on the tumor microenvironment—specifically on macrophages—rather than on tumor cells themselves. This supports the positioning of ALK5 inhibitors among the few therapeutic strategies, alongside immune checkpoint inhibitors, that act predominantly by targeting stromal rather than tumor cell-intrinsic pathways.

While our study establishes ANG as a central modulator of the therapeutic activity of ALK5 inhibition, several important questions remain. The source of elevated ANG levels in PDAC patients and the factors regulating its expression in tumor cells warrant further investigation. Additionally, we have recently identified additional stromal-mediated mechanisms of acquired resistance to ALK5 inhibitors, such as autotaxin, an enzyme secreted by inflammatory cancer-associated fibroblasts (CAFs) in response to TGFβ signaling inhibition [23]. Future studies exploring the spatial interactions between these mediators of intrinsic and acquired resistance within the tumor microenvironment using technologies such as multiplex imaging or spatial transcriptomics could provide valuable insights to refine patient selection and enhance the efficacy of TGFβ-targeted therapies.

In conclusion, this study identifies the novel ANG–EGFR–TGFβ–TNFα signaling cascade in TAMs as a critical driver of therapeutic resistance in PDAC. This axis represents a promising target for therapeutic intervention, with combination strategies involving TGFβ pathway inhibitors potentially enhancing chemotherapy efficacy in tumors with high ANG expression. By providing both mechanistic insight and clinical validation, our findings support the use of Ang expression as a predictive biomarker to stratify patients with PDAC most likely to benefit from such combinatorial approaches.

## Supplementary information

Supplementary Methods and Figures

## Data Availability

All data supporting the results are available upon request from the corresponding author.
