# Supplementary material for: EGFR ligand Angiogenin predicts response to ALK5 inhibition in pancreatic cancer via a TNF-α paracrine axis in tumor-associated macrophages
Source: Oncogene. 2026 Apr 14;45(20):1901–13. doi: 10.1038/s41388-026-03774-0 (PMC13167465; doi:10.1038/s41388-026-03774-0)
Supplement: Supplementary file 1 — Supplementary Methods and Figures [file 41388_2026_3774_MOESM1_ESM.docx]

**SUPPLEMENTARY INFORMATIONS**

**SUPPLEMENTARY MATERIALS AND METHODS**

**Study population**. This study focused on patients enrolled in the phase 2 of the H9H-MC-JBAJ clinical trial, a global, two-part investigation assessing the oral administration of galunisertib in combination with gemcitabine. The initial phase of the study consisted of a multicenter, open-label, non-randomized dose-escalation trial. The subsequent phase employed a randomized, double-blind, two-arm design in a 2:1 ratio, comparing galunisertib plus gemcitabine to gemcitabine with placebo.

Eligible participants were required to meet the following criteria: (1) a histologically or cytologically confirmed diagnosis of pancreatic ductal adenocarcinoma (PDAC), either locally advanced (Stage II or III) or metastatic (Stage IV), for which surgical resection with curative intent was not feasible. Patients who had previously undergone curative surgery for PDAC could be included upon documented disease progression. Those who had received adjuvant treatment with gemcitabine-based chemotherapy or chemoradiotherapy were eligible if such therapy had been completed at least three months prior to enrollment; (2) the presence of measurable or non-measurable disease, as defined by RECIST version 1.1; (3) age ≥18 years, regardless of sex; (4) adequate baseline organ function, including hematologic (absolute neutrophil count ≥1.5 × 10⁹/L, platelet count ≥100 × 10⁹/L, hemoglobin ≥9 g/dL), hepatic (bilirubin ≤1.5 × upper limit of normal [ULN], AST, ALT, and ALP ≤2.5 × ULN), and renal (serum creatinine ≤1.5 × ULN) parameters; (5) an Eastern Cooperative Oncology Group (ECOG) performance status of 0 to 2; (6) absence of moderate to severe cardiovascular disease; and (7) exclusion of individuals with endocrine tumors of the pancreas or ampullary carcinomas. All patients provided written informed consent before participation, and the protocol was approved by the ethics committee of the Azienda Ospedaliera Universitaria Integrata di Verona. All procedures involving human participants, tissues, or data were conducted according to the principles of good clinical practice, applicable laws and regulations, the Council for International Organizations of Medical Sciences International Ethical Guidelines, and the Declaration of Helsinki.

**TCGA-PAAD RNA-seq processing, ANG stratification, macrophages infiltration.** RNA-seq data from the TCGA pancreatic adenocarcinoma (TCGA-PAAD) cohort were from the Genomic Data Commons using the TCGAbiolinks package. Analyses were conducted separately according to the TCGA classification of tumor annotation (primary vs metastasis), with patients filtered using PCA-based Mahalanobis distance outlier detection on normalized transcriptomic profiles (p < 0,001). Macrophage score was estimated from bulk RNA-seq using TIMER (1), while M2-macrophage infiltration using CIBERSORT (2). Filtered TPM values were used as input via the immunedeconv R packages (3), and scores were extracted for each sample. The median expression value of ANG was used for patients’ stratification. Scores from TIMER and CIBERSORT were then compared between groups using a one-sided Wilcoxon rank-sum test.

**Compounds and treatments.** Recombinant Angiogenin (rAng) (Cat. No.CSB-EP001703MO, Cusabio Technology LLC) and recombinant Tnfα (rTnfα) (Cat. No. 410-MT, R&D System) were used for 48h at 250 ng/mL (rAng) or 200 ng/mL (rTnfα), respectively. Gemcitabine (Cat. no.S1149), etanercept (Cat. no. HY108847), galunisertib (Cat. No. S2230), Cytochalasin D (Cat. no. HY-N6682), Rhosin hydrochloride (Cat. no. HY-12646), and ETH-1864 (Cat. No. HY-16659) were purchased from MedChemExpress. For in vitro experiments, all compounds were dissolved in 100% dimethyl sulfoxide (DMSO) and used at the following concentrations: 0.78nM-200nM gemcitabine, 5µM galunisertib, 5μg/ml etanercept, 2.5μM cytochalasin D, 10μM rhosin hydrochloride or 15µM ETH-1864. For in vivo treatments, gemcitabine was resuspended in saline solution and administered intraperitoneally at 75 mg/Kg concentration once/week, while galunisertib was resuspended in hydroxyethylcellulose (HEC) (0.25%) and administered via oral gavage at 100 mg/kg concentration twice/day.

**Cell cultures**. Pancreatic cancer cells were kindly provided by Dr. Paola Cappello (University of Turin, Italy) and Prof. Vincenzo Bronte (University of Verona). Cells were isolated by tumors developed in LSL-Kras^G12D/+^; p53^R172H/+^; PdxCre^tg/+^ (KPC) or LSL-Kras^G12D/+^; PdxCre^tg/+^ (KC) mice. RAW 264.7 cells were purchased from ATCC. All cells were cultured in Dulbecco’s modified Eagle’s medium (DMEM) (Euroclone, Milan, Italy) containing 10% fetal bovine serum (FBS) (Euroclone), 1% penicillin-streptomycin (PS) solution (Gibco) and 1% Glutamine (glu) (Gibco) at 37°C in a 5% CO_2_ incubator. All cells were regularly tested by PCR to exclude Mycoplasma contamination.

Bone-marrow derived macrophages (BMDM) were isolated by flushing femurs of adult C57BL6/J mice and culturing the resulting cells in RPMI containing 10% FBS, 1% PS and 1% glu supplemented with 50ng/mL recombinant m-CSF (Miltenyi Biotec) for 7 days, and then polarized for 48h with IFN-γ (50 ng/mL, Miltenyi Biotec) or Tgfβ (20 ng/mL, Bio-techne).

Co-culture experiments were performed using a hydrophobic silicon barrier in ø100 mm Petri dishes. 1x10^6^ BMDM or 1x10^6^ RAW264.7 were plated in half of the dish with 6x10^5^ PDAC cells, and let to attach overnight. After cutting the barrier, cells were allowed to communicate for 48h in low serum conditions (DMEM 1% FBS) in the absence or presence of treatments.

**Quantification of the effect of treatments.** To evaluate the response of PDAC cell lines to the combination of gemcitabine plus galunisertib, the conditioned medium (CM) from co-cultures was filtered and used for treatments. 1x10^3^ cells were seeded in 96-well plates and treated for 72h with the CM containing DMSO or increasing concentrations of gemcitabine, in presence or absence of 5µM galunisertib. When indicated, 5µg/ml etanercept or 200 ng/ml rTnfα were combined. Crystal violet was used to measure cell viability using a plate reader (Victor X4, PerkinElmer). Mean EC50 values and 95% CIs were calculated using GraphPad Prism software (GraphPad Software Inc).

**Angiogenin depletion from conditioned medium**. 3x10^5^ FC1245^Scr^ or FC1245^shAng^ cells were seeded in p60, let to adhere overnight and then starved for 48h. Conditioned media (CM) were then filtered using a 0.22μm filter, and subjected to immunoprecipitation by overnight incubation with 50 μl of protein A/G PLUS-agarose beads (Santa Cruz Biotechnology, #sc-2003) and 2.5 μg of mouse anti-Angiogenin (Santa Cruz Biotechnology, #sc-74528) or normal mouse IgG (Santa Cruz Biotechnology, #sc-2025), followed by centrifugation at 10000×g for 15 min at 4°C. The bound fraction was eluted after boiling for 10 min at 95 °C in 2× Laemmli sample buffer (Bio-Rad, catalog no.: 1610747), and analyzed by Western blot using mouse anti-Angiogenin (Cat. 14017.7, Invitrogen). The unbound fractions from IP-IgG or IP-Ang CM were collected, filtered, and used to stimulate BMDM for 48h. Western blot analysis of unbounded CM using mouse anti-Angiogenin (Cat. 14017.7, Invitrogen) was also performed to confirm Ang depletion in the IP-Ang samples compared to those immunoprecipitated with the IgG control.

**T cell suppression assay.** Murine T cells were isolated by grinding spleens of healthy C57BL/6J mice through a 70 μm cell strainer, and red blood cells lysed with Red Blood Cell Lysis Solution (Cat. 130-094-183, Miltenyi Biotec). T cells were then purified using Pan T Cell Isolation Kit II mouse (Cat. 130-095-130, Miltenyi Biotec) according to the manufacturer’s instructions, and then activated for 48h with 10 μg/mL anti- mouse anti-CD3ε and 2 μg/mL soluble anti-mouse anti-CD28 antibodies using the T Cell Activation/Expansion Kit mouse (Cat. 130-093-627, Miltenyi Biotec).

Evaluation of T cell suppression was performed as in (4). Briefly, activated T cells were co-cultured at 1:5 ratio with normal BMDM or with BMDM pre-stimulated with rAng for 48h. Co-culture experiments were performed in BMDM cell growth media (RPMI, 10% FBS, 1% P/S, 1% glutamine). The percentage of dead cells was detected by flow cytometry after staining with CD3-PE Cy7 (REA641), CD4-FITC (REA604), CD8a-BV421 (53-6.7), and the Viobility Fixable dye, all from Miltenyi Biotec. Subsequently, cells were fixed, permeabilized with the Inside Stain Kit (Cat. 130-090-477, Miltenyi Biotec), and stained with anti-IFNγ-APC (clone REA638, Miltenyi Biotec) to evaluate intracellular IFNγ production.

**Plasmids, siRNA silencing and lentiviral production.** Egfr siRNA (sc-29302), Plexin B2 siRNA (sc-45423) and control siRNA (sc-37007) were purchased from Santa Cruz Biotechnology and transfected according to manufacturer’s instructions.

Lentiviruses for gene knockdown were produced in HEK-293T cells by co-transfecting lentiviral vector, dR8.74 packaging plasmid (Addgene #22036) and pMD2.G envelope plasmid (Addgene #12259). shRNA vectors used were: pLKO.1-puro (scramble, LV-c; Addgene #8453), pLKO.1-puro-shAng.1 (targeting sequence 5′-GCAGGGTTCAGACATGTTGTT-3′ and pLKO.1-puro-shAng.2 (targeting sequence 5′- TGATGAAGAGAAGAAGCCTAA-3′). When unspecified, gene silencing was performed by co-infection of PDAC cells with both Ang-targeting lentiviruses. Lentiviruses for gene overexpression were produced in HEK-293T by co- transfection of pLenti-GIII-CMV vector (Cat. No. 49645064, Applied Biological Materials Inc.) or pLenti-GIII-CMV-Ang (Cat. No. 11885064, Applied Biological Materials Inc.), with dR8.74 packaging (Addgene #22036) and pMD2.G envelope vectors (Addgene #12259).

**Immunofluorescence.** 15.000 BMDM cells were seeded on positively charged glass slides (Thermo Fisher Scientific), fixed for 30 min with 4% paraformaldehyde (PFA) (Thermo Fisher Scientific) and 4% sucrose (Sigma) solution, washed with PBS and permeabilized with PBS/BSA 1%/Triton 0.3% solution for 10 min. Cell cytoskeleton F-actin was labeled with Alexa Fluor® 555 Phalloidin (#8953, Cell Signaling Technology, Danvers, MA, USA) for 15 min. Next, BMDM were washed with PBS, cell nuclei stained with DAPI (#4083, Cell Signaling Technology, Danvers, MA, USA), and slides closed with glass coverslips using as mounting medium a 50% glycerol solution. Images were acquired at 60X magnification with an FLUO VIEW 400-EVIDENT fluorescence microscope. For each condition, a range of 10 random fields per slide was analyzed.

**EGFR activation and dimerization assay.** 1x10^6^ BMDM were seeded in 60mm dishes in complete medium to allow them to adhere overnight, serum-starved for 16h and stimulated with 400 ng/ml of murine Egf (#67074, Cell Signaling Technology), 1 μg/ml of murine recombinant Angiogenin (Cat. CSB-EP001703MO, Cusabio Technology LLC) on ice for 1h. Cells were then crosslinked with 3 mM BS3 (Sigma-Aldrich, Cat.no. 82436-77-9) for 20 min on ice and quenched with 250 mM glycine for 5 min at 4 °C. Pellets were lysed in ice-cold lysis buffer (140 mM NaCl, 10 mM EDTA, 10% glycerol, 1% NP-40, and 20 mM TRIS-HCl pH 8) supplemented with protease and phosphatase inhibitors, and supernatant was collected as whole cell extract (WCE) after centrifugation at 16,000 × g at +4 °C for 20 min. 50 μg proteins were resolved on a 6% SDS-polyacrylamide gel, transferred overnight onto a nitrocellulose membrane and incubated with anti-Egfr antibody (Santa Cruz Biotechnology, sc-373746).

**Rho/Rac GTPase activity.** The active GTPase pull-down assays were performed according to the manufacturer’s instructions for the active RAC1 detection kit (Cat. No. 8815, Cell Signaling Technology) and active Rho Detection Kit (Cat. No. 8820, Cell Signal Technology), with samples treated with GTPγS (positive control) and GDP (negative control). The immunoprecipitated materials and total protein samples were then prepared for immunoblot analysis.

**RNA extraction and real-time qPCR.** RNA extraction was performed using PureLink RNA Mini Kit (Invitrogen) and quantified by using Nanodrop 8000. After DNase I treatment (Roche Diagnostics), 1 μg of RNA was reverse transcribed with High-Capacity RNA-to-cDNA™ Kit (Applied Biosystems). Quantitative real-time PCR was carried out at 60°C using PowerUp SYBR Green Master Mix (Applied Biosystems) on a QuantStudio3 (Applied Biosystems). Primers were designed by using Primer3. Primer sequences are reported in Supplementary Table 1.

**Chromatin Immunoprecipitation (ChIP).**

For ChIP experiments, the RAW264.7 cell line was used instead of BMDM according to the 3R practices, given the requirement of high number of cells. Briefly, 4x10^6^ RAW264.7 were fixed with 1% formaldehyde for 15 min, and fixation stopped by addition of 125 mM glycine for 5 min. Cells were lysed in Farnham lysis buffer (5 mM PIPES pH 8, 85 mM KCl, 0.5% NP-40) supplemented with protease inhibitors for 8 min. Nuclei were collected by centrifugation and lysed in nuclear lysis buffer (1% SDS, 10 mM EDTA, 50 mM Tris-HCl pH 8) supplemented with protease inhibitors for 20 min. Chromatin was sonicated with an ultrasonic bath (8 cycles, 30 sec ON, 30 sec OFF) to an average size of 200–600 bp, diluted with Dilution Buffer (10 mM Tris-HCl pH 8, 2 mM EDTA, 140 mM NaCl, 1% Triton X-100, 0.1% SDS) and incubated overnight with 20 μl dynabeads protein G and 3 μg of mouse anti-Smad2 (phospho-S465) (#ab216482, Abcam) or normal rabbit IgG (#2729, Cell Signaling Technologies). Immunocomplexes were washed with increasing salt concentrations, DNA eluted at 65°C with 1% SDS and recovered with PureLink RNA mini kit (Cat. 12183018A, Invitrogen). qPCR was carried out at 60°C using PowerUp SYBR Green Master Mix (Thermo Fisher Scientific) on a QuantStudio3 (Applied Biosystems). Promoter sequence of Tnf has been obtained at UCSC Genome Browser GRCm38/mm10 (chr17: 35203907-35206007). Primer sequences are reported in Supplementary Table 1.

**Protein extraction, western blot and immunoprecipitation.** For western blot, cells were lysed in cold RIPA buffer (Cat. ab156034, Abcam) supplemented with protease and phosphatase inhibitors (#5872, Cell Signaling Technologies) and centrifuged at 20000xg for 20min at 4°C. Protein quantification was performed using the Pierce BCA Protein Assay kit (Thermofisher Scientific). Equal amounts of protein were resolved by SDS-polyacrylamide gel electrophoresis, transferred onto nitrocellulose membranes, and incubated for 1h in Every blot blocking buffer (Cat. No 12010020, Bio-Rad) at room temperature.

For cytoplasmic-nuclear extraction, cells were lysed using the NE-PER™ Nuclear and Cytoplasmic Extraction kit (Cat. 78835, ThermoFisher Scientific) according to manufacturer instructions. Hsp90 and histone H3 were used to quantify cytoplasm or nucleus, respectively.

For protein immunoprecipitation experiments, 500 μg whole cell extract was incubated overnight with 50 μl of protein A/G PLUS-agarose beads (Santa Cruz Biotechnology, #sc-2003) and 2.5 μg of rabbit anti-TGFβR1 (Abcam, # ab31013), or normal rabbit IgG Isotype Control (Cell Signaling Technologies, #3900). Proteins were eluted after boiling for 10 min at 95 °C in 2× Laemmli sample buffer (Bio-Rad, catalog no.: 1610747).

Blotted membranes were developed by using Immobilon Western Chemiluminescent HRP Substrate (Merck Millipore) and imaged with UVITEC Alliance Q9 Advanced (UVITEC Cambridge). Primary antibodies are reported in Supplementary Table 2.

**Enzyme-linked immunosorbent assay (ELISA).** ELISA assays were performed with mouse Angiogenin ELISA Kit (Novus Biologicals, Cat. No. NBP2-68208), mouse Tnfα ELISA Kit (R&D System Cat. no MTA00B), and mouse Tgfβ ELISA Kit (Novus Biologicals, Cat. no NBP1-92671). Conditioned media or plasma samples from mice were diluted before use following manufacturer’s instructions. Human Angiogenin (Cat. No. DAN00) and human TNFα (Cat. No. DTA00D) ELISA kits from R&D System were used to assess ANG and TNFα levels in plasma samples from patients. The optical density was determined with microplate reader iMark (Bio-Rad) at 540 nm.

Nf-κB binding activity was measured using the ELISA-based TransAM Nf-κB Activation kit (Cat.40596, Active Motif, Carlsbad, San Diego) according to manufacturer’s instruction. The optical density was determined with microplate reader iMark (Bio-Rad) at 450 nm, and corrected for background levels.

**Immunohistochemistry.** Immunohistochemistry (IHC) and hematoxylin and eosin (H&E) were performed on murine pancreatic sections of formalin-fixed paraffin-embedded specimens cut at 5 μm interval. After antigen retrieval with citrate buffer pH 6.0 (Leica Biosystems), tissues were blocked with 2.5% BSA blocking solution (Vector laboratories) for 1h and incubated with primary antibodies against CD68 or CD206 overnight at 4°C, or stained with H&E (cat. no H-3502, Vector Laboratories). Slides were developed using ImmPACT DAB substrate peroxidase HRP substrate (Vector Laboratories) following manufacturer’s instructions, and staining evaluated blindly. Primary antibodies are reported in Supplementary Table 2.

**Flow cytometry.** Tumors were dissociated using Tumor dissociation kit mouse (Cat. no 130-096-730, Miltenyi Biotec) according to manufacturer's instructions. Cells were collected, filtered by 70μm cell strainer and centrifuged for 5 min at 1000 rpm. Cell pellets (1 × 10^6^) were resuspended in 100μL of PBS and incubated for 15 min at 4°C with a panel antibody cocktail of CD45 APC-Vio770 (REA737), CD11b APC (REA592), F4/80 PE (REA126), CD3 PE-Vio770 (REA641), CD8a VioBlue (53-6.7), CD178 PE-Vio 615 (REA1171), Ly6G VioBlue (REA526), Ly6C FITC (REA796), NK1.1 FITC (REA1162), all from Miltenyi Biotec. T reg staining was performed with MACS Inside Stain kit (Cat. 1 30-090-477) with the panel antibody cocktail of CD45 APC-Vio770 (REA737), CD4 PerCP-Vio700 (REA604), CD25 APC (REA568), FoxP3 PE (REA788), all from Miltenyi Biotec. Macrophage phenotype was evaluated using CD204 PE-Vio770 (C068C2) for M2 TAMs, and CD86 VioBright B515 (REA1190) for M1 TAMs, respectively. The Fixable die Viobility SYTOX Blue (Invitrogen) was used to gate for live cells. Acquisition was performed at the Centro Piattaforme Tecnologiche (CPT) facility of the University of Verona using Becton Dickinson LSR-Fortessa X-20, and samples analyzed blindly using FlowJo software (TreeStar Inc., Ashland, USA).

**In vivo orthotopic transplantations.** 5-6 weeks old C57BL/6J female with body weight ranging from 21 to 25g were purchased from Charles River Laboratories. Murine PDAC cells were resuspended in a PBS:Matrigel solution (1:1) at a concentration of 2.5X10^5^ cells per 40 μl/injection. On day 0, mice were anesthetized by exposure to isoflurane and injected orthotopically into the pancreas parenchyma. For in vivo treatments, mice bearing pancreatic cancer were randomly allocated into 8 groups (n=12) to receive galunisertib (100 mg/kg) via oral gavage twice/day, gemcitabine (75 mg/kg) intraperitoneally once/week, or their vehicles. For Kaplan-Meier survival analysis, 6 mice from each group were euthanized by cervical dislocation when tumors reached the ethical cutoff volume 1500 mm^3^, and included in the survival curve. For flow-cytometry and ELISA, 6 mice from each group were euthanized by cervical dislocation at the end of 4-weeks treatment, peripheral blood was collected by retro-orbital sinus puncture, and tumors were collected for ex vivo blinded analysis. Sample-size was estimated using G power analysis. Mice were maintained at the animal facility of the University of Verona, in a pathogen-free and temperature-controlled environment with 12h light and dark cycles, housed in plastic cages and fed ad libitum. Research involving animals was conducted according to the relevant guidelines and regulations and has been approved by the Italian Ministry of Health (authorization no. 299/2022-PR from Ministry of Health, prot. C46F4.29).

**REFERENCES**

1. Li B, Severson E, Pignon JC, Zhao H, Li T, Novak J, et al. Comprehensive analyses of tumor immunity: implications for cancer immunotherapy. Genome Biol. 2016 Aug 22;17(1):174. doi: 10.1186/s13059-016-1028-7.
2. Newman AM, Liu CL, Green MR, Gentles AJ, Feng W, Xu Y, et al. Robust enumeration of cell subsets from tissue expression profiles. Nat Methods. 2015 May;12(5):453-7. doi: 10.1038/nmeth.3337.
3. Sturm G, Finotello F, Petitprez F, Zhang JD, Baumbach J, Fridman WH, et al. Comprehensive evaluation of transcriptome-based cell-type quantification methods for immuno-oncology. Bioinformatics. 2019 Jul 15;35(14):i436-i445. doi: 10.1093/bioinformatics/btz363.
4. Giammona A, De Vellis C, Crivaro E, Maresca L, Amoriello R, Ricci F, et al. Tumor-derived GLI1 promotes remodeling of the immune tumor microenvironment in melanoma. J Exp Clin Cancer Res. 2024 Aug 2;43(1):214. doi: 10.1186/s13046-024-03138-0.

**SUPPLEMENTARY FIGURES AND TABLES**

**Supplementary Figures**

**Figure S1:** Immunophenotyping of orthotopic PDAC models.

**Figure S2:** Tumor-derived Ang correlates with M2-TAMs infiltrate in PDAC models.

**Figure S3:** Ang depletion from conditioned medium dampens the M2-skewing of BMDM in vitro.

**Figure S4:** Ang induces M2-skewing of RAW264.7 macrophages in in vitro co-culture models.

**Figure S5:** The M2 polarization state induced by Ang is associated with increased cellular elongation.

**Figure S6:** Ang enhances the ability of BMDM to suppress T cells.

**Figure S7:** EGFR acts as the functional receptor for Ang.

**Figure S8:** Inhibition of TGFβ in macrophages sensitizes Ang-high PDAC cells to gemcitabine-based chemotherapy.

**Figure S9:** TAMs-derived Tnfα is the mediator of TGFβ signaling in sustaining chemoresistance of Ang-high PDAC cells.

**Figure S10:** Gating strategy.

**Supplementary Tables**

**Table S1.** List of primers used in this study.

**Table S2.** List of antibodies used in this study.

**Figure S1: Immunophenotyping of orthotopic PDAC models.** **(A)** Single-cell suspensions were gated for physical parameters, including forward scatter (FSC) for size, and side scatter (SSC) for granularity. Representative sample FACS plots of FC1245-bearing C57BL/6J mice are shown. **(B)** Flow cytometry analysis showing immune cell infiltration in FC1245, FC1199, B6KPC, MET610, DT6606 and DT4313 orthotopic tumors. Data are expressed as mean ± s.d. (n=6).

**Figure S2: Tumor-derived Ang correlates with M2-TAMs infiltrate in PDAC models.** **(A)** Pearson’s correlation between Ang mRNA expression and the infiltration of TAMs in tumors from C57BL6/J PDAC models (n=24). **(B)** Pearson’s correlation between secreted Ang and the infiltration of Cd86^+^/Cd204^-^ M1-like TAMs in tumors from C57BL6/J PDAC models (n=24). **(C)** Gating strategy. TAMs were gated based on CD45+/Cd11b+/F4/80+ positivity, and evaluated for the expression of Cd86 and Cd204 (M1 TAMs: Cd86+/Cd204-; M2 TAMs: Cd86-/Cd204+). Sample FACS plots of DT4313^NTC^ and DT4313^Ang^-bearing C57BL/6J mice are shown. **(D-F)** Representative images of paraffin sections from DT4313^NTC^, DT4313^Ang^, FC1245^Scr^ and FC1245^shAng^ orthotopic tumors stained with Cd68 or Cd206 antibodies (D) and relative quantifications (E,F). Scale bar=60 μm. Data are expressed as mean ± s.d. (n=6). P values were calculated by two-tailed unpaired Student's t test. *p<0.05; **p<0.01; ***p<0.001.

**Figure S3: Ang depletion from conditioned medium dampens the M2-skewing of BMDM in vitro.** **(A)** Representative Western blot of Angiogenin in FC1245^Scr^ or FC1245^shAng^ cells. Hsp90 was used as loading control. **(B)** Conditioned media (CM) from FC1245^Scr^ or FC1245^shAng^ cells were immunoprecipitated (IP) with either IgG (IP-IgG) or anti-Angiogenin antibody (IP-Ang), as denoted by Ang detection in agarose beads (bound fraction). **(C)** Western blot of Ang in the CM showing angiogenin content previously depleted by IP (unbound fraction). **(D)** qPCR analysis of M1 (Inos2, Cd86) and M2 (Arg1, Mrc1) markers in BMDM left unstimulated or stimulated for 48h with the unbound fraction of FC1245^Scr^ or FC1245^shAng^ CM previously immunoprecipitated with IP-IgG or IP-Ang. Data are shown as mean ± s.d (n=3). P values were calculated using Anova with Tukey’s correction. *p<0.05; **p<0.01; ***p<0.001.

**Figure S4: Ang induces M2-skewing of RAW264.7 macrophages in in vitro co-culture models.** **(A)** Schematic representation of the co-culture technique used to culture RAW264.7 cell line with PDAC cell lines. (**B**) qPCR analysis of M2 (Arg1, Mrc1) and M1 (Inos2, Cd86) markers in RAW264.7 as well as single cultures or co-cultured with DT4313 or FC1245 transduced as indicated. Data are shown as mean ± s.d (n=4). P values were calculated by ANOVA and Tukey’s test. *p<0.05; **p<0.01; ***p<0.001. (**C**) Representative Western blot of pSmad2 and Smad2 in RAW264.7 as single cultures or co-cultured with DT4313 or FC1245 transduced as indicated. Densitometry measurements of pSmad2 protein bands relative to total Smad2 from 3 independent experiments are shown.

**Figure S5: The M2 polarization state induced by Ang is associated with increased cellular elongation.** **(A)** Representative immunofluorescence images of BMDMs cultured 48 hrs in RPMI medium and treated with Tgf-β1, Ifn-γ, rAng or their control vehicles (CTR). BMDMs were stained with Phalloidin and counterstained with DAPI. Scale bar = 20 μm. **(B)** qPCR analysis of M1 (*Nos2, Cd86*) and M2 (*Arg1, Mrc1*) markers in BMDM left unstimulated or stimulated with Tgfβ, Ifnγ or rAng. Data are shown as mean ± s.d (n=4). P values were calculated by ANOVA and Tukey’s test. ***p<0.001.

**Figure S6: Ang enhances the ability of BMDM to suppress T cells.**  **(A)** Representative gating strategy. Single-cell suspensions were gated for physical parameters, including forward scatter (FSC, size) and side scatter (SSC, granularity). Purified T cells were gated based on CD45^+^/Cd3^+^/Cd4^+^ or CD45^+^/Cd3^+^/Cd8^+^ positivity. (**B, C**) Flow cytometry analysis of T cells co-cultured for 48h with BMDM (1:5 ratio), left unstimulated or pre-stimulated for 24h with rAng (**B**). The percentage of dead cells was determined by Viobility staining gated on CD3^+^ or CD3^+^/CD8^+^ cells (**C**). P values were calculated using two-tailed unpaired Student's t test.

**Figure S7: EGFR acts as the functional receptor for Ang.** (**A**) qPCR analysis of *Egfr* and *Plxb2* in BMDM, FC1245, FC1199, B6KPC, DT6606, MET-610 and DT4313 cell lines. Data are shown as mean ± s.d (n=3). (**B**) qPCR analysis of *Nos2*, *Cd86,* *Arg1*, *Mrc1* in BMDMs left unstimulated or stimulated with rAng, and treated with 2.5μM gefitinib for 48h. Data are shown as mean ± s.d (n=3). (**C**) qPCR analysis of *Nos2*, *Cd86,* *Arg1*, *Mrc1* in BMDM left unstimulated or stimulated with rAng, and transiently transfected with shPlexinB2 for 48h. Data are shown as mean ± s.d (n=3). (**D**) Densitometry measurements of pErk1/2 protein bands relative to total Erk1/2 and of pStat3 protein bands relative to total Stat3 from at least 3 independent experiments as shown in Fig.3E. (**E**) Densitometry measurements of pErk1/2 protein bands relative to total Erk1/2, and of pSmad2 protein bands relative to total Smad2 from 4 independent experiments as shown in Fig.3I. P values were calculated by ANOVA and Tukey’s test. *p<0.05; **p<0.01; ***p<0.001.

**Figure S8: Inhibition of TGFβ in macrophages sensitizes Ang-high PDAC cells to gemcitabine-based chemotherapy.** Dose-response curves in FC1245 or DT4313 after 72h of treatment with increasing doses of gemcitabine in combination with 2.5μM galunisertib as single cultures (A), as co-cultured with BMDMs that were pre-treated with 2.5μM galunisertib (B) or as co-cultured with BMDMs and then treated with gemcitabine in combination with 2.5μM galunisertib. Tables indicates EC50 values and 95% CIs of gemcitabine. GEM: gemcitabine; GAL: galunisertib; CM: conditioned medium.

**Figure S9: TAMs-derived Tnfα is the mediator of TGFβ signaling in sustaining chemoresistance of Ang-high PDAC cells. (A)** qPCR of *Tnf* in FC1245^Scr^, FC1245^shAng^, DT4313^NTC^ or DT4313^Ang^ as single culture or in coculture with BMDMs and treated for 72hrs with 2.5μM galunisertib. Data are expressed as mean ± s.d. (n=3). P values were calculated using ANOVA and Tukey’s test. **(B)** Representative Western blot of p-Nf-κB and Nf-κB in FC1245^Scr^, FC1245^shAng^, DT4313^NTC^ or DT4313^Ang^ as single cultures or co-cultured with BMDMs, treated as indicated for 48h. Gapdh was used as loading control. Densitometry measurements of pNf-κB protein bands relative to total Nf-κB from 4 independent experiments are shown. **(C)** Schematic representation of the in vitro treatment schedule. **(D-G)** Dose-response curves of FC1245 (C,D) or DT4313 (E,F) transduced as indicated and co-cultured with BMDMs, after 72h of treatment with gemcitabine alone or in combination with 2.5μM galunisertib, in presence or absence of 20 ng/mL recombinant Tnfα (rTnfα). Tables indicates EC50 values and 95% CIs of gemcitabine. GAL: galunisertib; rTnfα: recombinant Tnfα.

**Figure S10: Gating strategy.** Single-cell suspensions were gated for physical parameters, including forward scatter (FSC, size) and side scatter (SSC, granularity). TAMs were gated based on CD45+/Cd11b+/F4/80+ positivity and evaluated for the expression of Cd86 and Cd204 (M1 TAMs: Cd86+/Cd204-; M2 TAMs: Cd86-/Cd204+). Sample FACS plots of FC1245^Scr^, FC1245^shAng^, DT4313^NTC^ or DT4313^Ang^ -bearing C57BL/6J mice treated with gemcitabine as single agent or in combination with galunisertib are shown. GEM: gemcitabine; GAL: galunisertib.

**Supplementary Table 1. List of primers used in this study.**

| **Primer** | **Sequence (5’ to 3’)** |
| --- | --- |
| Ang Fwd | CCAGGCCCGTTGTTCTTG |
| Ang Rev | CCAGAGTGGGAGGGATCACA |
| Tgfb1 Fwd | TTACCTTGGTAACCGGCTGC |
| Tgfb1 Rev | AGCCCTGTATTCCGTCTCCT |
| Hprt Fwd | AGTCCCAGCGTCGTGATTAG |
| Hprt Rev | GCCTCCCATCTCCTTCATGA |
| Arg1 Fwd | CTGAGCTTTGATGTCGACGG |
| Arg1 Rev | TCCTCTGCTGTCTTCCCAAG |
| iNos2 Fwd | CCCCGCTACTACTCCATCAG |
| iNos2 Rev | CCACTGACACTTCGCACAAA |
| Tnf Fwd | GTGCCTATGTCTCAGCCTCT |
| Tnf Rev | CTGATGAGAGGGAGGCCATT |
| Mrc1 Fwd | ATGGGCAACATCGAGCAGAA |
| Mrc1 Rev | AAACCAATGCAACCCAGTGC |
| Cd86 Fwd | CCGGATGGTGTGTGGCATAT |
| Cd86 Rev | TGAGCAGCATCACAAGGAGG |
| Tnf prom A Fwd | TCACATCCCCACAGTCTCCA |
| Tnf prom A Rev | TCCCATGTCTGTCCCTCCTT |
| Tnf prom B Fwd | GGGTGGAGAGAGATGAGGGT |
| Tnf prom B Rev | CCAATCCGTATGACTCCCCG |

**Supplementary Table 2. List of antibodies used in this study.**

| **Antibody** | **Source** | **Cat. No.** | **Dilution** |
| --- | --- | --- | --- |
| Angiogenin | Invitrogen | 14017.7 | 1:1000 (WB) |
| Angiogenin | Santa Cruz Technologies | #sc-74528 | 2.5 μg (IP) |
| Egfr | Abcepta | AM7628b | 1:1000 (WB) |
| phospho-Egfr Tyr 1068 (D7A5) | Cell Signaling Technology | 3777 | 1:1000 (WB) |
| Plexin B2 | Santa Cruz Technologies | sc-373930 | 1:500 (WB) |
| phospho-Tyr-1000 | Cell Signaling Technology | 8954 | 1:1000 (WB) |
| TgfβRI | Abcam | ab31013 | 2.5 μg (IP) |
| Nf-kB (C22B4) | Cell Signaling Technology | 4764 | 1:1000 (WB) |
| phosho-Nf-kB Ser 536 (93H1) | Cell Signaling Technology | 93H1 | 1:1000 (WB) |
| phospho-Smad2 Ser 465/467 (138D4) | Cell Signaling Technology | 3108 | 1:1000 (WB)  1:200 (IHC) |
| Smad2 | Cell Signaling Technology | 3103 | 1:1000 (WB) |
| Tgfβ1 | RayBiotech | 102-11520 | 1:1000 (WB) |
| phospho-Erk1/2 Thr 202/Tyr 204 (20G11) | Cell Signaling Technology | 4376 | 1:1000 (WB) |
| Erk1/2 (L34F12) | Cell Signaling Technology | sc-13119 | 1:1000 (WB) |
| phospho-Stat3 Tyr 705 | Cell Signaling Technology | 9131 | 1:1000 (WB) |
| Stat3 (124H6) | Cell Signaling Technology | 9139 | 1:1000 (WB) |
| Actin | Santa Cruz Technologies | sc-47778 | 1:5000 (WB) |
| Hsp90 | Santa Cruz Technologies | sc-13119 | 1:10000 (WB) |
| Gapdh | Thermo Fisher Scientific | G9545 | 1:50000 (WB) |
| RhoA | Cell Signaling Technology | 2117 | 1:1000 (WB) |
| Rac1 | Cell Signaling Technology | 8631 | 1:500 (WB) |
| CD68 | R&D system | MAB101141 | 1:100 (IHC) |
| CD206 | Abcam | ab64693 | 1:1000 (IHC) |

WB: Western blotting; IHC: Immunohistochemistry; IP: Immunoprecipitation.
